# Exploring the Potential of Calebin‐A in Targeting Obesity‐Related Genes and Pathways

**DOI:** 10.1111/jcmm.71244

**Published:** 2026-06-12

**Authors:** Ali Mahmoudi, Ali Saeedi‐Boroujeni, Sercan Karav, Prashant Kesharwani, Amirhossein Sahebkar

**Affiliations:** ^1^ Department of Basic Medical Sciences, Faculty of Medicine Abadan University of Medical Sciences Abadan Iran; ^2^ Department of Molecular Biology and Genetics Canakkale Onsekiz Mart University Canakkale Turkey; ^3^ Next‐Generation Translational Nanomedicine Laboratory, Department of Pharmaceutical Sciences Dr. Harisingh Gour Vishwavidyalaya Sagar Madhya Pradesh India; ^4^ University Institute of Pharma Sciences, Chandigarh University Mohali Punjab India; ^5^ Biotechnology Research Center, Pharmaceutical Technology Institute Mashhad University of Medical Sciences Mashhad Iran; ^6^ Centre for Research Impact and Outcome, Chitkara College of Pharmacy Chitkara University Rajpura Punjab India; ^7^ Center for Innovation and Inclusive Research Sharda University Greater Noida Uttar Pradesh India; ^8^ Applied Biomedical Research Center, Basic Sciences Research Institute Mashhad University of Medical Sciences Mashhad Iran

**Keywords:** bioinformatics, calebin‐A, gene expression, molecular docking, obesity, pathway analysis

## Abstract

Obesity is a global health crisis affecting millions, associated with metabolic disorders such as type 2 diabetes and cardiovascular disease. Calebin‐A, a bioactive compound derived from *Curcuma* species, has shown promise in managing obesity and its complications. This study utilized bioinformatics tools to explore the molecular mechanisms of Calebin‐A in obesity. Transcriptomic data from obese and normal omental adipose tissue (GSE286454) were analysed, identifying 317 differentially expressed genes (DEGs). Functional enrichment analysis indicated a notable engagement of lysosomal activity, immune response, cell migration, axon guidance and apoptosis pathways. A STRING‐based protein–protein interaction network revealed nine hub genes through a composite centrality score. Among these, CTSB, CTSZ, CTSA, GRN and TUBB exhibited upregulation and were prioritized for subsequent analysis. External validation (GSE59034; 16 obese vs. 16 controls) corroborated the consistent upregulation of CTSB, CTSZ, GRN and CTSA. Target prediction analysis identified 443 potential targets for Calebin‐A, with pathway‐level overlap suggesting a convergence on immune, lysosomal and cytoskeletal processes. Molecular docking studies indicated favourable binding affinities (−5.2 to −7.1 kcal/mol), with CTSZ demonstrating the most robust interaction. A 100 ns molecular dynamics simulation validated structural stability and indicated favourable binding free energy (MM‐PBSA Δ*G* ≈ −110 kJ/mol). Results suggest that Calebin‐A targets genes and proteins involved in energy balance and inflammation, offering insights into its anti‐obesity potential. These findings provide a foundation for experimental validation and therapeutic development.

## 1 Introduction

Obesity, characterized by excessive adiposity that impairs health, is a global epidemic affecting approximately 650 million individuals [1, 2]. It significantly elevates the risk of numerous metabolic disorders, notably type 2 diabetes (T2D), with about two‐thirds of those with obesity also experiencing prediabetes, a condition marked by insulin resistance and beta‐cell dysfunction that increases the lifetime risk of T2D by up to 70% [3, 4]. Beyond T2D, obesity is associated with cardiovascular disease, metabolic‐dysfunction associated steatotic liver disease (MASLD), osteoarthritis and obstructive sleep apnea [5]. This profound health burden underscores the urgency of effective management strategies and therapeutic targets for obesity and its comorbidities [6, 7, 8].

Management of obesity primarily relies on lifestyle interventions, including diet, exercise and behavioural modifications, which offer diverse health benefits but typically achieve only modest weight loss (up to 10%). Long‐term maintenance remains elusive, with approximately 80% of lost weight regained within 5 years due to physiological adaptations such as reduced resting metabolic rate and heightened appetite, potentially driven by altered orexigenic and anorexigenic signals [9]. Although 5%–10% weight loss is clinically beneficial, greater reductions may be necessary to achieve remission of certain obesity‐related complications [10, 11]. Bariatric surgery provides substantial weight loss (25%–30%) and sustained outcomes but is limited by scalability and patient hesitancy due to perceived postoperative risks [12, 13, 14].

The aetiology of obesity extends beyond environmental factors, such as high‐caloric diets and sedentary lifestyles, to include a significant genetic component. Twin and family studies demonstrate heritability estimates ranging from 40% to 75%, with higher concordance rates for obesity in monozygotic versus dizygotic twins, highlighting a strong genetic predisposition [15]. Advances in genome‐wide association studies (GWAS) and next‐generation sequencing (NGS) have elucidated both monogenic and polygenic causes of obesity. Monogenic obesity arises from variants in single genes, including leptin (*LEP*), leptin receptor (*LEPR*), proopiomelanocortin (*POMC*) and melanocortin 4 receptor (*MC4R*), while polygenic obesity involves multiple genetic loci [16]. Syndromic obesity, often accompanied by developmental delays, includes conditions such as Prader–Willi and Bardet‐Biedl syndromes [16]. These genetic insights are pivotal for developing targeted, personalized therapeutic approaches.

Calebin‐A, a potent bioactive compound, was first extracted from *Curcuma longa* [17] and subsequently identified in *Curcuma caesia* [18]. Extensive research highlights the therapeutic potential of these plants in modern medicine, particularly for their prophylactic roles in combating oxidative stress [19], inflammation [20] and malignancies [21, 22, 23]. As a secondary metabolite, Calebin‐A exhibits a robust safety profile, even at elevated doses [24]. It is characterized as an aglycone glucuronidated metabolite with a serum half‐life ranging from approximately 1–3 h, predominantly cleared through non‐renal pathways [19]. Calebin‐A in a study indicated that it inhibits adipogenesis, enhances lipolysis and reduces hepatic steatosis, aiding metabolic syndrome [25].

Over the past decade, bioinformatics tools have transformed drug discovery by accelerating target identification in biomedical research. This interdisciplinary field combines computer science and molecular biology to process extensive biological datasets, such as those from genomics, transcriptomics and proteomics [26]. Numerous bioinformatic‐ or deep learning‐based platforms have been developed to facilitate the prediction and analysis of disease‐gene, drug‐disease and protein–protein associations. These platforms are designed with the overarching goal of enhancing our understanding of disease pathogenesis, predicting therapeutic targets and identifying potential drug candidates [27, 28, 29, 30]. Notably, high‐throughput genomic and transcriptomic analyses and protein–protein interaction (PPI) networks are widely used to detect disease‐specific targets and potential mechanisms of action of drugs/drug candidates in various diseases [31, 32, 33, 34]. The principles of network pharmacology have been widely utilized to identify mechanisms of action for a broad spectrum of natural products across various disorders [34, 35, 36, 37]. Moreover, virtual screening and molecular docking provide time‐ and cost‐efficient alternatives to conventional experimental methods for investigating drug‐disease connections [38, 39, 40, 41, 42]. Specifically, molecular docking enables the assessment of how small molecules interact with macromolecular targets by evaluating their binding site compatibility [43, 44]. Although the therapeutic potential of Calebin‐A has been partially explored, its in silico mechanisms related to obesity and associated protein targets remain largely unstudied. Additionally, a detailed computational analysis connecting Calebin‐A to diabetes‐related targets has not yet been published.

This study aims to utilize bioinformatics tools to identify potential molecular targets of Calebin‐A in obesity and to explore its mechanisms of action through gene expression analysis, PPI networks, molecular docking and molecular dynamics simulation.

## 2 Methods

### 2.1 Data Acquisition and Preprocessing

The transcriptomic data were obtained from the Gene Expression Omnibus (GEO) under the accession number GSE286454. The dataset comprised whole transcriptome sequencing data from the greater omentum adipose tissue of five obese individuals and five healthy controls. The raw count matrix was downloaded and preprocessed in R to ensure data integrity. Duplicate gene names were addressed by summing counts for technical replicates, and rows with empty or NA gene names were removed. The metadata, including sample conditions (obese vs. normal), were loaded and matched to the count matrix.

### 2.2 Differential Expression Analysis

Differential gene expression analysis was performed using the DESeq2 package in R. The DESeqDataSet was constructed from the count matrix and metadata, with the design formula set to compare gene expression between obese and normal samples. Low‐count genes were filtered out (retaining genes with row sums ≥ 10 counts), and the DESeq2 pipeline was executed to estimate size factors, dispersion and perform negative binomial generalized linear model fitting. Results were extracted with a significance threshold of adjusted *p*‐value (*p*adj) < 0.05 and absolute log2 fold change (|log2FC|) > 0.9.

### 2.3 Functional Enrichment Analysis of DEGs

#### 2.3.1 Gene Ontology and Pathway Enrichment Analysis

To systematically elucidate the biological importance of differentially expressed genes (DEGs) identified in the context of obesity compared to normal omental adipose tissue, we performed an extensive functional enrichment analysis. The gene symbols of significant DEGs (*p*adj < 0.05, |log2FoldChange| > 0.9) were examined using the Enrichr web platform (https://maayanlab.cloud/Enrichr) [45], utilizing the following gene set libraries: Gene Ontology (GO) Biological Process, Cellular Component, Molecular Function, KEGG 2026, Reactome Pathways and WikiPathways 2024. For each library, we obtained tab‐separated results that included pathway terms, adjusted *p*‐values (Benjamini Hochberg) and counts of gene overlap.

#### 2.3.2 Protein–Protein Interaction Network Construction and Analysis

The PPI network was constructed utilizing the STRING database (version 12.0) with a medium confidence threshold (combined score ≥ 400), concentrating solely on the 318 significant DEGs. The network was visualized and analysed using the R packages igraph and tidygraph. The statistical significance of the network was evaluated through a hypergeometric enrichment test, which compared the number of observed interactions among the query proteins to the expected number based on the complete STRING human interactome (score ≥ 400); a *p*‐value < 0.05 signifies a non‐random, biologically relevant network. Network statistics, including total proteins, total interactions, average connections, network diameter, characteristic path length and clustering coefficient, were computed to characterize the global properties of the PPI network. The network was simplified to remove self‐loops and duplicate edges. Node attributes, including degree centrality, betweenness centrality, closeness centrality and eigenvector centrality, were calculated to identify key topological features. Differential expression data (log2FoldChange and adjusted *p*‐values) were integrated into the network to annotate nodes as ‘Upregulated’ and ‘Downregulated’.

To identify hub proteins, we utilized a multi‐attribute centrality approach. For each node, we computed four centrality measures: degree (the number of direct connections), betweenness (the fraction of shortest paths that pass through the node), closeness (the inverse of the average distance to all other nodes) and eigenvector centrality (the influence based on the importance of neighbours). Each measure was normalized to a range of 0–1 using min–max scaling. A composite centrality score was established as the sum of the four normalized values. Proteins with a composite score in the top 8th percentile were classified as hubs. This multi‐dimensional strategy mitigates bias towards high‐degree but peripheral nodes and captures various aspects of topological centrality.

#### 2.3.3 Cluster Analysis Using MCODE

To identify functionally relevant modules within the PPI network, the MCODE algorithm was employed. The network was clustered using the cluster_fast_greedy function in igraph, which optimizes modularity to detect densely connected subgraphs. Nodes were assigned to clusters based on their membership in these subgraphs. The resulting clusters were visualized with a force‐directed layout, and cluster‐specific properties (size and connectivity) were analysed.

#### 2.3.4 In Silico Validation

To assess the reproducibility of our findings, we employed the independent dataset GSE59034 (subcutaneous adipose tissue; 16 obese vs. 16 never obese controls). Differential expression data were sourced from GEO2R (limma). For the hub proteins selected for molecular docking, we did not impose a predefined log2 fold change threshold for external validation. Instead, we evaluated reproducibility based on direction consistency (the same sign of logFC) and statistical significance (adj.*p* < 0.05) in the independent dataset.

#### 2.3.5 Identification of Calebin‐A Targets and Obesity‐Related Genes

The 2D/3D structure and SMILES notation of Calebin‐A were retrieved from PubChem (CID: 637429). Potential protein targets were predicted using multiple computational platforms (SwissTargetPrediction, SEA Search Server, TargetNet and PPB Browser), which employ machine learning algorithms (e.g., Naïve Bayes, deep neural networks) trained on chemical fingerprints and biological activity data from ChEMBL. Obesity‐related genes were extracted from the GEO dataset (GSE286454) after differential expression analysis (adjusted *p* < 0.05, |log2FC| > 0.9). Gene symbols were standardized using org.Hs.eg.db in R, resolving ENSEMBL IDs to official symbols.

#### 2.3.6 Venn Diagram and Functional Enrichment Analysis

The overlap between Calebin‐A targets and obesity genes was visualized using a pairwise Venn diagram (VennDiagram R package). To evaluate functional convergence beyond the restricted direct gene overlap (six genes), we analysed the enriched pathways (adjusted *p* value < 0.05) derived from the obesity and Calebin A studies. We identified overlapping pathways by aligning pathway names that were significantly enriched in both gene sets. For each overlapping pathway, we retrieved the adjusted *p* value and gene count from both the obesity and Calebin A findings. All subsequent processing—which included the extraction of pathway terms, the computation of –log10(adjusted *p* values), and the creation of overlap tables—was conducted in R (version 4.5) utilizing the dplyr, tidyr and ggplot2 packages.

### 2.4 Molecular Docking

#### 2.4.1 Target Proteins and Ligand Preparation

The two‐dimensional configuration of Calebin‐A was sourced from the PubChem database, a publicly accessible repository for chemical substances that includes structures of both small and large molecules, along with biological assays and physicochemical characteristics [46]. The three‐dimensional structures of significant hub proteins related to obesity were acquired from the Protein Data Bank (PDB), which serves as an open‐access archive for experimentally determined protein structures derived from methods such as X‐ray crystallography and nuclear magnetic resonance spectroscopy. The PDB is an essential resource that contains 3D structural information on biomacromolecules, enabling in silico investigations into ligand‐receptor interactions at the atomic scale [47]. We employed molecular docking simulations to investigate the potential direct binding interactions between Calebin‐A and the hub proteins.

#### 2.4.2 Molecular Docking Process

Before initiating the docking procedure, we refined the protein structures utilizing Dockamon software (version 1.0). The docking process was executed with PyRx version 1.0, which facilitates the simultaneous docking of various small molecules against macromolecular targets [48]. To improve the precision of our results, we adjusted the exhaustiveness parameter to 80. The results of the docking simulations were evaluated based on the binding energy (ΔGbind) between the Calebin‐A and the target proteins. Additionally, the binding predictions were utilized. These predictions incorporate three complementary scoring functions: (i) consensus score (the average of classical functions) [49], (ii) SVM‐based score [50], and (iii) random forest‐based score (RF Score v2) [51]. These machine learning models are trained on experimentally validated ligand‐protein complexes and yield independent affinity estimates (pK = −log10(Kd)). Finally, we used Discovery Studio Visualizer 4.5 to examine the hydrogen‐bonding interactions occurring between the ligands and the target proteins.

#### 2.4.3 Molecular Dynamics Simulation

To investigate the dynamic behaviour of the protein–ligand complexes, molecular dynamics (MD) simulations were performed using the NAMD software package. The initial setup and subsequent visualization of the systems were managed with VMD. Each complex was solvated in a TIP3P water environment, and charge neutrality was ensured by the addition of counter ions. Protein parameters were described by the CHARMM36 force field, while ligand‐specific parameters were obtained from the CHARMM General Force Field (CGenFF).

The simulation protocol involved an initial energy minimization phase of 10,000 steps. Following minimization, the systems were gradually heated to 310 K at constant volume. A 1 ns equilibration period was conducted under the NPT ensemble to ensure stable system conditions. Subsequently, a 100 ns production run was performed with a time step of 2 fs. Periodic boundary conditions were applied throughout the simulation to mimic bulk solvent conditions, and electrostatic interactions were calculated using the Particle Mesh Ewald (PME) method.

Analysis of the simulation trajectories in VMD focused on assessing structural stability and flexibility. The overall stability of the systems was monitored through the calculation of backbone Root Mean Square Deviation (RMSD) values. Furthermore, binding free energies were estimated using the Molecular Mechanics with the Poisson‐Boltzmann and Surface Area solvation (MM‐PBSA) approach. For this calculation, distinct trajectories were generated for the complex, the receptor alone, and the ligand alone. The energy contributions, encompassing van der Waals forces, electrostatic interactions, polar solvation energy and nonpolar Surface Area (SASA) energy, were then evaluated.

## 3 Results

### 3.1 Differential Expression Analysis

The DESeq2 analysis identified 317 significantly differentially expressed genes between obese and normal omentum adipose tissue (*p*adj < 0.05, |log2FC| > 0.9). Among these, 209 genes were upregulated and 108 were downregulated in obesity.

**FIGURE 1:**
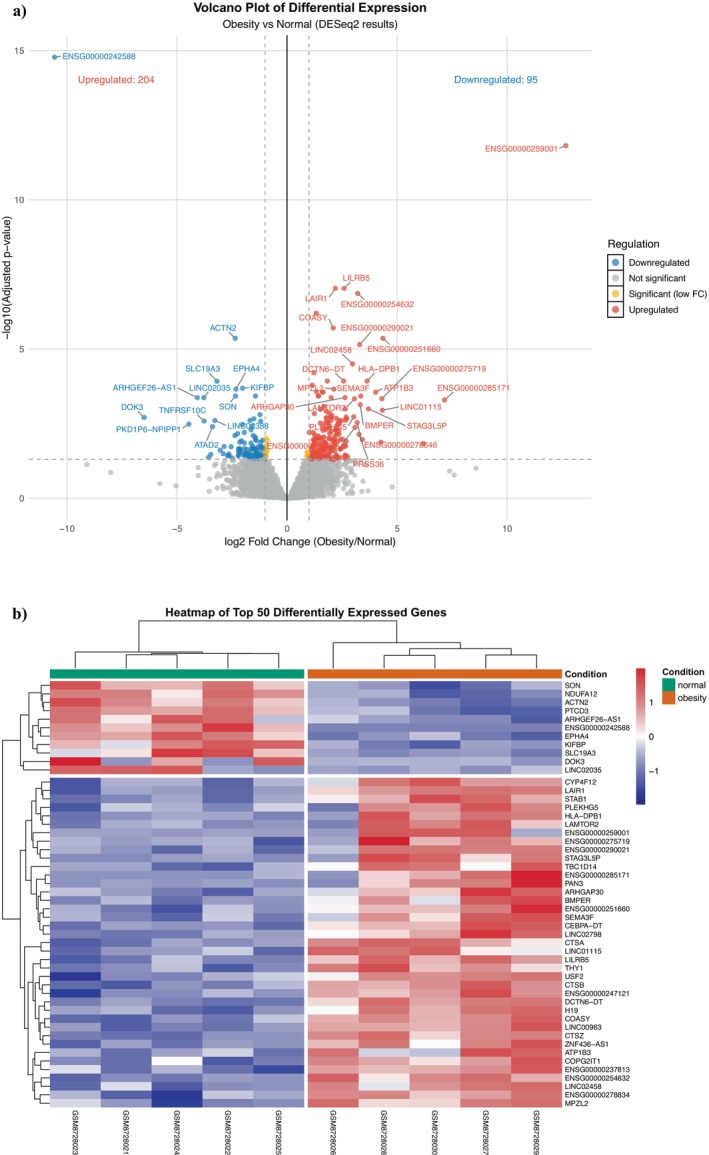
Heatmap illustrating the differentiation of samples with differentially expressed genes (DEGs) in GSE286454 (a), alongside a Volcano Plot that showcases significant fold changes in gene expression, where downregulated genes are marked in blue and upregulated genes in red (b).

The plot (Figure 1a) revealed a clear separation of DEGs, with extreme fold changes and high statistical significance (*p*adj < 1e−10). The heatmap (Figure 1b) showed distinct clustering of obese and normal samples, with genes such as LAIR1, LILRB5 and HLA‐DPB1 forming prominent upregulated clusters, whereas ACTN2 and SLC19A3 clustered among downregulated genes.

### 3.2 Functional Enrichment Analysis of Differentially Expressed Genes

**FIGURE 2:**
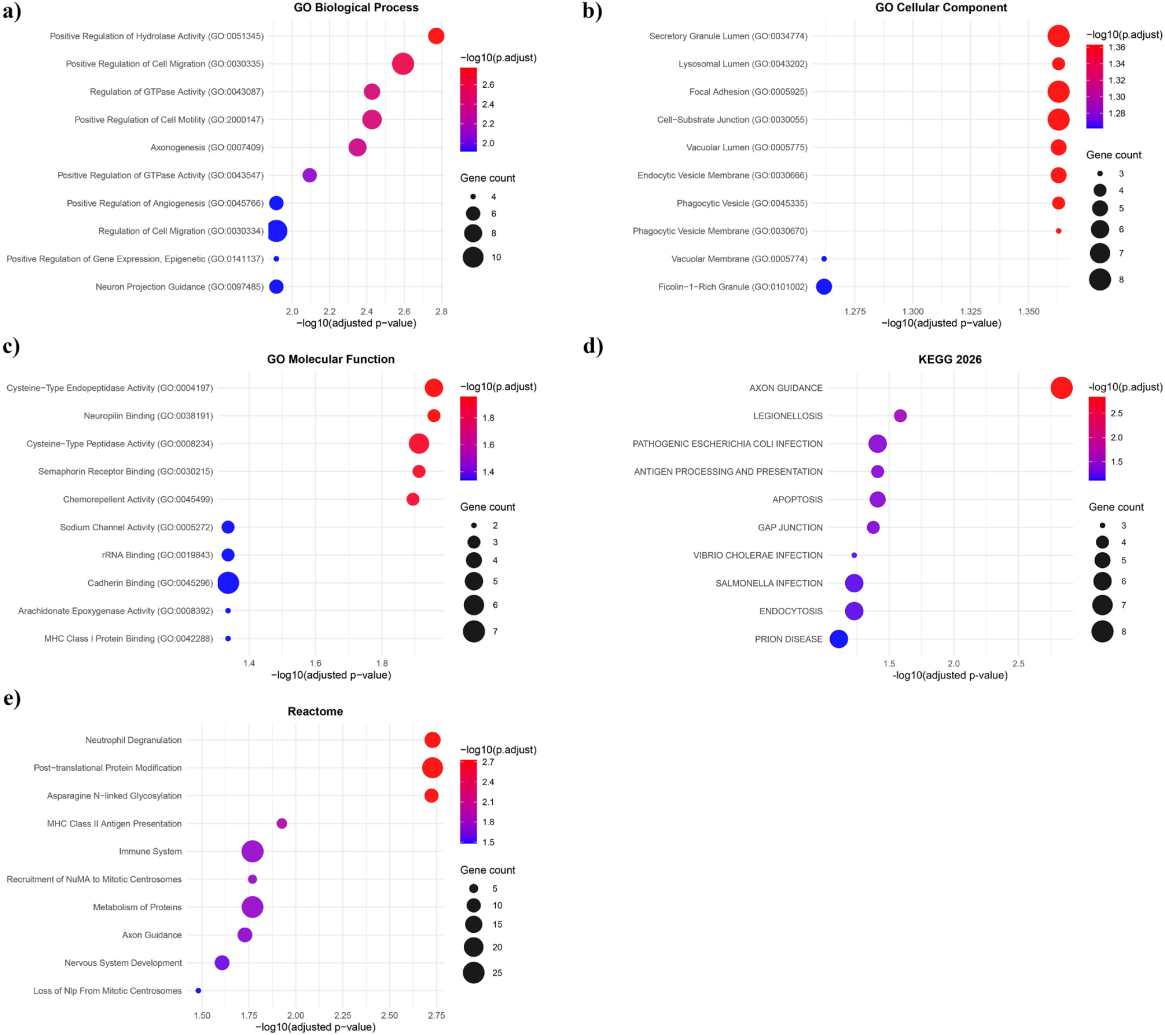
Analysis of functional profiling for differentially expressed genes, illustrating enrichment across biological process‐level (a), cellular component (b), molecular function enrichment (c), KEGG pathways (d) and Reactome pathways (e).

The functional enrichment analysis of the 317 significant differentially expressed genes (DEGs) focuses solely on protein‐coding genes, identifying key biological processes, molecular functions, cellular components and pathways linked to obesity‐related changes in omental adipose tissue (Figure 2).

The Gene Ontology (GO) Biological Process analysis indicated a significant enrichment of terms associated with cell migration, GTPase signalling and axonal development. Particularly, the positive regulation of hydrolase activity (GO:0051345, adj.*p* = 1.69 × 10^−3^), regulation of GTPase activity (GO:0043087, adj.*p* = 3.74 × 10^−3^), and axonogenesis (GO:0007409, adj.*p* = 4.47 × 10^−3^) were among the most significantly overrepresented processes, implying altered intracellular signalling and neuro‐immune interactions in obese adipose tissue (Figure 2a).

The GO Cellular Component analysis revealed an enrichment of compartments associated with vesicular trafficking and the extracellular matrix. The secretory granule lumen (GO:0034774, adj.*p* = 4.34 × 10^−2^), lysosomal lumen (GO:0043202, adj.*p* = 4.34 × 10^−2^) and focal adhesion (GO:0005925, adj.*p* = 4.34 × 10^−2^) were significantly enriched, suggesting a dysregulation of lysosomal secretory pathways and cell matrix adhesion (Figure 2b).

The GO Molecular Function analysis underscored the significance of cysteine‐type endopeptidase activity (GO:0004197, adj.*p* = 1.10 × 10^−2^), cysteine‐type peptidase activity (GO:0008234, adj.*p* = 1.23 × 10^−2^) and semaphorin receptor binding (GO:0030215, adj.*p* = 1.23 × 10^−2^), reinforcing the involvement of cathepsin‐like proteases and axon guidance molecules in obesity (Figure 2c).

KEGG pathway analysis revealed a significant enrichment in axon guidance (hsa04360, adj.*p* = 1.47 × 10^−3^), antigen processing and presentation (hsa04612, adj.*p* = 3.88 × 10^−2^) and apoptosis (hsa04210, adj.*p* = 3.88 × 10^−2^), thereby establishing a connection between immune activation and neuronal signalling with adipose tissue dysfunction (Figure 2d).

Reactome pathway analysis identified enriched terms associated with innate immunity and protein metabolism. Notably, neutrophil degranulation (adj.*p* = 1.87 × 10^−3^), MHC class II antigen presentation (adj.*p* = 1.19 × 10^−2^) and axon guidance (adj.*p* = 1.87 × 10^−2^) were found to be overrepresented, aligning with the findings from GO and KEGG analyses (Figure 2e). In summary, these enrichment findings underscore the intersection of inflammatory, lysosomal and neuro guidance pathways in the development of obesity.

### 3.3 PPI Network Topology and Hub Proteins

**FIGURE 3:**
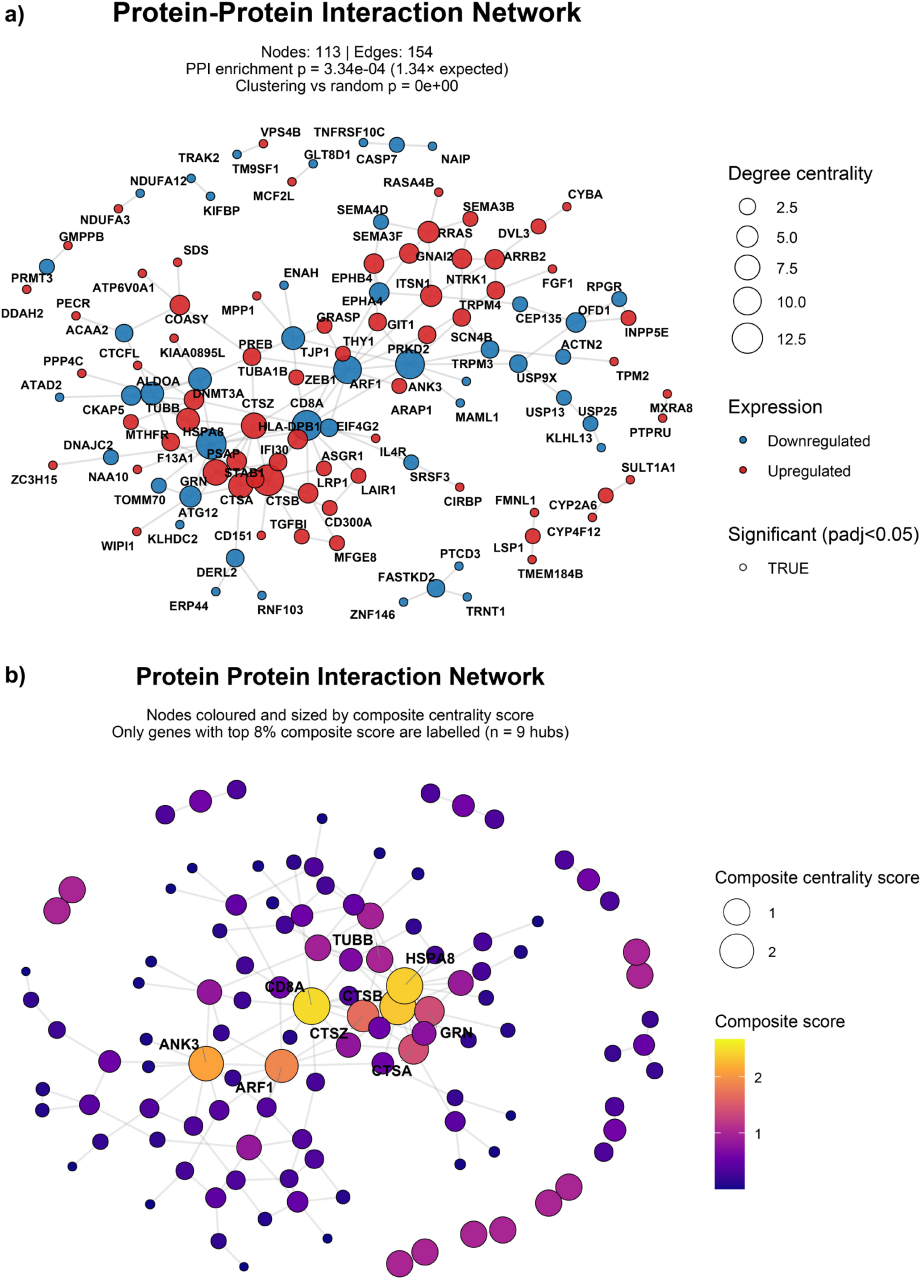
(a) Visualization of the PPI network constructed from up and down regulated of GSE286454 (*p*adj < 0.05, |log2FC| > 0.9). Node size indicates degree centrality, and colour saturation represents up/down expression. (b) Subnetwork of hub proteins and their first neighbours. The subgraph highlights the top 8% of proteins by composite centrality score. Hub nodes are coloured and sized by composite centrality score.

The refined PPI network consisted of 113 proteins and 154 interactions (Figure 3a). The network demonstrated an average degree of 2.73, a diameter of 9, and a characteristic path length of 4.04. The clustering coefficient was measured at 0.18, which was significantly greater than that of random networks of comparable size (permutation test *p* = 0.0009). Most notably, a hypergeometric enrichment test against the complete STRING human background indicated that the interactions observed among the DEGs were significantly higher than what would be anticipated by chance (PPI enrichment *p* = 3.34 × 10^−4^), thereby confirming that the network is biologically relevant and not merely a random configuration.

Hub proteins, such as HSPA8 (Degree = 12, Betweenness = 691.62), CTSB (Degree = 12, Betweenness = 407.44) and ANK3 (Degree = 11, Betweenness = 1126.29), were identified as critical nodes with high connectivity and centrality (Table 1, Figure 3b). These hubs were predominantly involved in key cellular processes, including protein folding, metabolic regulation and immune response.

**TABLE 1:** Hub genes diagnosed in the PPI Network: Degree, betweenness centrality, composite score, logFC and adjusted *p*‐values in obesity.

Protein	Full gene name	Degree	Betweenness	Closeness	Eigenvector	Composite_Score	log2FoldChange	*p*adj	Regulation
ANK3	Ankyrin 3	11	1.23E+03	4.30E‐03	2.70E‐01	2.11E+00	−1.73E+00	3.79E‐02	Downregulated
ARF1	ADP‐Ribosylation Factor 1	10	9.32E+02	4.20E‐03	3.69E‐01	1.88E+00	−1.95E+00	6.21E‐03	Downregulated
CD8A	CD8A Molecule	12	1.11E+03	4.30E‐03	6.44E‐01	2.47E+00	−1.41E+00	3.80E‐02	Downregulated
CTSA	Cathepsin A	7	2.61E+02	3.50E‐03	6.94E‐01	1.41E+00	1.33E+00	2.66E‐04	Upregulated
CTSB	Cathepsin B	13	4.14E+02	4.00E‐03	1.00E+00	2.34E+00	1.22E+00	6.20E‐05	Upregulated
CTSZ	Cathepsin Z	8	3.98E+02	4.00E‐03	7.62E‐01	1.67E+00	1.33E+00	6.33E‐07	Upregulated
GRN	Progranulin	8	1.21E+02	3.50E‐03	6.98E‐01	1.38E+00	1.62E+00	9.80E‐03	Upregulated
HSPA8	Heat Shock Protein Family A (Hsp70) Member 8	12	7.67E+02	4.10E‐03	8.47E‐01	2.39E+00	‐1.53E+00	2.19E‐02	Downregulated
TUBB	Tubulin Beta Class I	6	1.18E+02	3.50E‐03	4.87E‐01	1.00E+00	1.49E+00	9.28E‐03	Upregulated

#### 3.3.1 Hub Proteins Based on Composite Centrality Score

Utilizing the composite score (the sum of normalized degree, betweenness, closeness and eigenvector centrality), we identified the top 8% of proteins as hubs (*n* = 9). The highest‐ranking hubs included CTSB (composite score = 2.34), HSPA8 (2.39), CD8A (2.47), ANK3 (2.11) and ARF1 (1.88). Among these, the upregulated hubs include CTSB, CTSZ (1.67), CTSA (1.41), GRN (1.38) and TUBB (1.00), which were chosen for subsequent molecular docking with Calebin A due to their representation as the most central and differentially expressed genes that exhibit consistent upregulation in obesity. Table 1 provides a summary of the composite scores, individual centrality metrics, and differential expression of the leading hubs.

### 3.4 Cluster Analysis and Functional Modules

**FIGURE 4:**
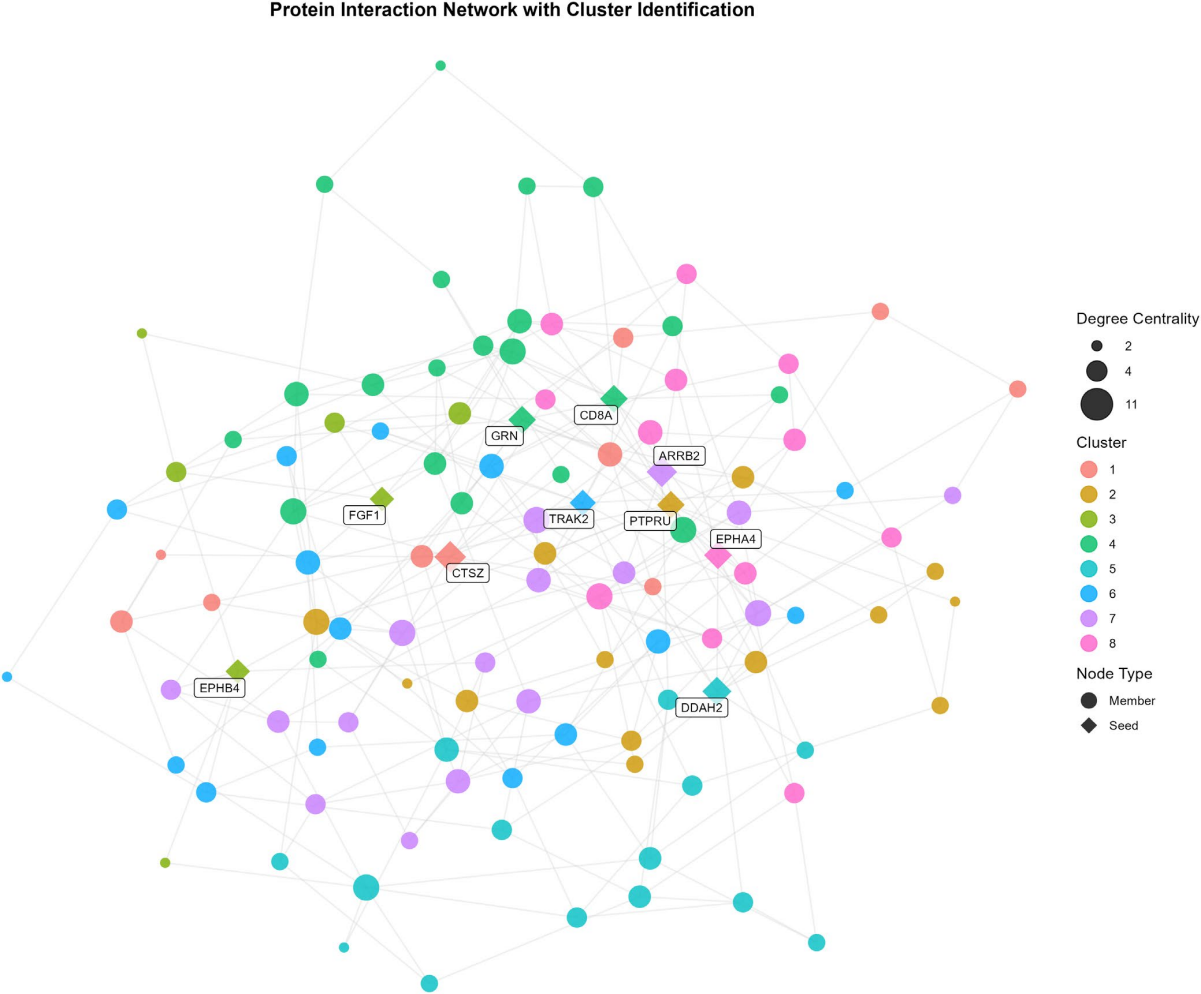
The identification of eight distinct clusters within the PPI network associated with obesity patients was conducted through MCODE analysis. Visualization techniques were employed, with nodes coloured according to their cluster affiliation and sized based on degree centrality. A colour gradient was utilized to distinguish between the clusters, while seed nodes were represented as triangles.

MCODE clustering analysis identified eight distinct modules (Clusters 1–8; Figure 4). Clusters that included the upregulated docking hubs were as follows: Cluster 2 (size 14, seed PTPRU degree 8) featuring CTSB (degree 7) and ITSN1 (degree 3); Cluster 4 (size 22, seeds CD8A and GRN, both degree 8) comprising GRN (degree 8), RRAS (degree 4), ATG12 (degree 4) and TUBA1B (degree 5); Cluster 6 (size 16, seed TRAK2 degree 7) containing TUBB (degree 6), ANK3 (degree 6) and CYBA (degree 6); and Cluster 8 (size 13, seed EPHA4 degree 8) with CTSA (degree 6), ALDOA (degree 5) and NTRK1 (degree 5).

### 3.5 Validation of Hub Genes in an Independent Dataset

**FIGURE 5:**
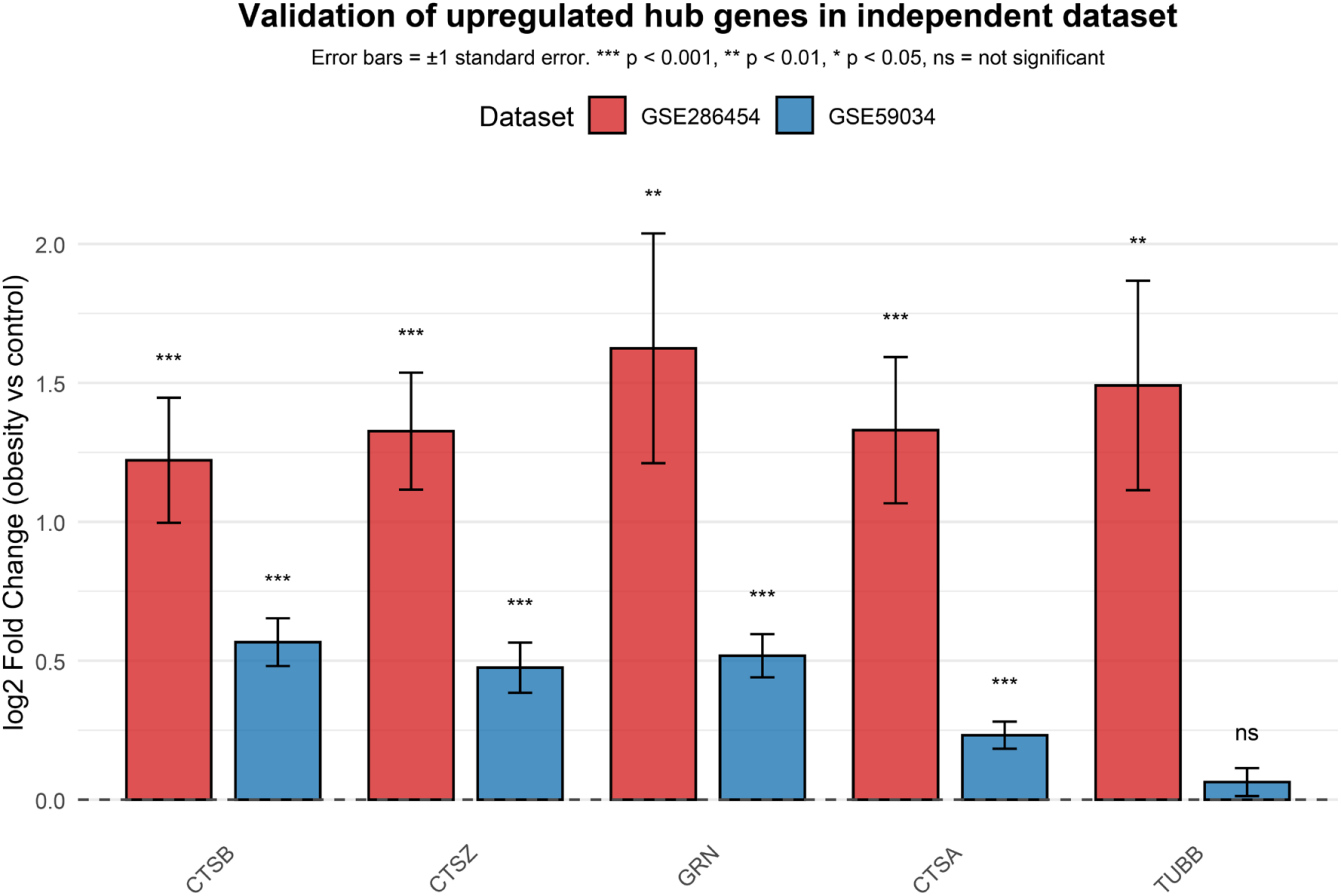
Validation of the five upregulated hub genes in an independent dataset (GSE59034). Bars represent log2 fold change (obesity vs. control) for each gene in the discovery (GSE286454, red) and validation (GSE59034, blue) datasets. Significance stars indicate adjusted *p*‐values: ***p < 0.001, **p < 0.01, *p < 0.05 (ns = not significant). Error bars represent ±1 standard error. All five genes (CTSB, CTSZ, GRN, CTSA, TUBB) show consistent up‐regulation in both datasets, with CTSB, CTSZ, GRN and CTSA reaching statistical significance in both. TUBB shows the same direction of change but does not reach significance in GSE59034 (*p* = 0.38).

To evaluate the reproducibility of our results, we confirmed the five upregulated hub genes (CTSB, CTSZ, GRN, CTSA, TUBB) utilizing the independent GSE59034 dataset (subcutaneous adipose tissue; 16 obese versus 16 never obese controls). All five genes displayed the same direction of change (up regulation) in both datasets (Table 2; Figure 5).

**TABLE 2:** Validation of five upregulated hub genes in the independent GSE59034 dataset.

Gene	log2FoldChange	*p*adj_286454	logFC	*p*adj_59034	Direction_consistent
CTSB	1.221587	6.20E‐05	0.567	1.52E‐05	TRUE
CTSZ	1.326422	6.33E‐07	0.475	0.000193	TRUE
GRN	1.624568	0.009795	0.518	1.35E‐05	TRUE
CTSA	1.330021	0.000266	0.232	0.000563	TRUE
TUBB	1.490656	0.009282	0.0634	0.378	TRUE

Four genes include TSB, CTSZ, GRN and CTSA, which were completely validated, showing consistent up regulation and statistical significance in both datasets (all adj.*p* < 0.01). Their log2 fold changes were similar across datasets (CTSB: 1.22 vs. 0.57; CTSZ: 1.33 vs. 0.48; GRN: 1.62 vs. 0.52; CTSA: 1.33 vs. 0.23), reinforcing their reliability as obesity‐related targets of Calebin A.

The fifth gene, TUBB, exhibited the same direction of change (up regulated) but did not achieve statistical significance in GSE59034 (logFC = 0.063, *p*adj = 0.378). This may indicate lower expression or greater variability of TUBB in subcutaneous adipose tissue compared to omental adipose, or the limited power of the discovery cohort (*n* = 5). However, several tubulin beta family members—including TUBB4B (logFC = 0.34, adj.*p* = 3.8 × 10^−4^), TUBB2A (logFC = 0.68, adj.*p* = 6.1 × 10^−4^), TUBB2B (logFC = 0.55, adj.*p* = 8.9 × 10^−4^) and TUBB6 (logFC = 0.24, adj.*p* = 0.013)—were significantly upregulated in GSE59034. This suggests that the tubulin beta family, rather than TUBB alone, is linked to obesity.

### 3.6 Overlap Between Calebin‐A Targets and Obesity Genes

**FIGURE 6:**
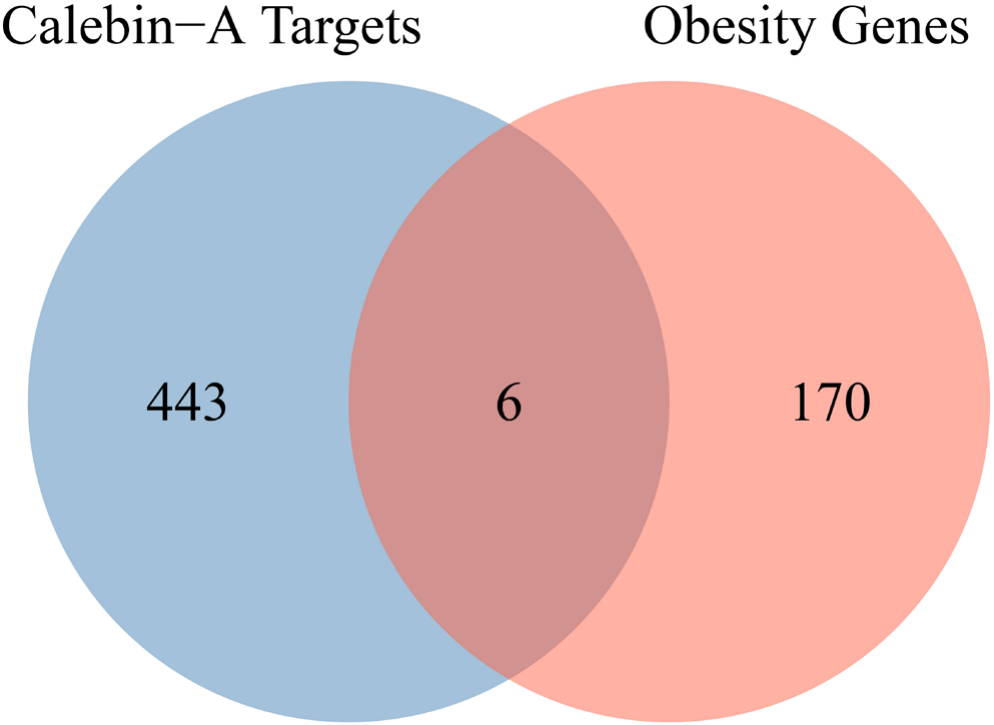
Intersection analysis of Obesity‐DEGs and Calebin‐A‐related targets using Venn diagram.

The Venn diagram analysis identified six overlapping genes between the 443 predicted targets of Calebin‐A and the 176 protein coding genes (after removing noncoding proteins by mapping to Entrez IDs) associated with obesity (Figure 6). These genes, namely EPH Receptor B4 (EPHB4), Neurotrophic Receptor Tyrosine Kinase 1 (NTRK1), NADH:Ubiquinone Oxidoreductase Subunit A3 (NDUFA3), NADH:Ubiquinone Oxidoreductase Subunit A12 (NDUFA12), Cytochrome P450 Family 2 Subfamily A Member 6 (CYP2A6) and Perilipin 1 (PLIN1), were selected for further investigation due to their critical roles in both Calebin‐A's molecular mechanisms and the pathophysiology of obesity.

### 3.7 Overlap of Pathway Enrichment Between Obesity DEGs and Calebin‐A Targets

**FIGURE 7:**
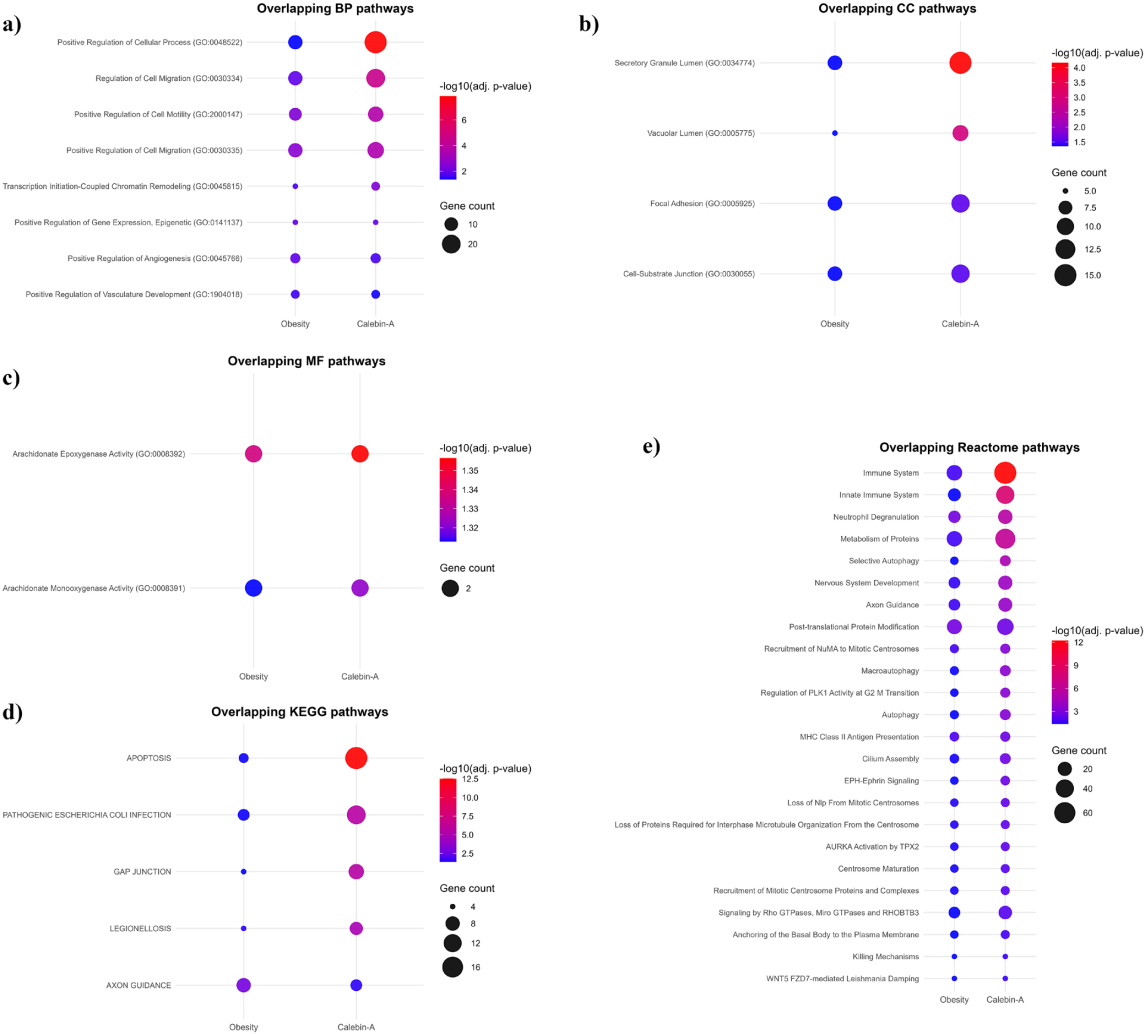
Enrichment analysis of overlap functional profiling for common pathways between differentially expressed genes and prediction calebin‐A targets. Biological process‐level (a), cellular component (b), molecular function enrichment (c), KEGG (d), and Reactome pathways (e).

To evaluate functional convergence beyond the restricted direct gene overlap (*n* = 6), we analysed the pathway enrichment profiles of the 317 obesity‐related differentially expressed genes (DEGs) and the 449 predicted target genes of Calebin A. We identified overlapping pathways for Gene Ontology (Biological Process, Cellular Component, Molecular Function), KEGG and Reactome (Figure 7).

The overlap in Biological Process included terms associated with cell migration and angiogenesis. Significantly, the positive regulation of cell migration (GO:0030335; obesity: adj.*p* = 2.55 × 10^−3^, Calebin A: adj.*p* = 2.76 × 10^−4^) and the regulation of cell migration (GO:0030334; obesity: adj.*p* = 1.22 × 10^−2^, Calebin A: adj.*p* = 2.70 × 10^−5^) were notably enriched in both datasets, indicating a common role in vascular and cellular plasticity (Figure 7a).

The overlap in Cellular Component indicated a shared involvement of lysosomal and extracellular compartments. The secretory granule lumen (GO:0034774; obesity: adj.*p* = 4.34 × 10^−2^, Calebin A: adj.*p* = 6.92 × 10^−5^) and vacuolar lumen (GO:0005775; obesity: adj.*p* = 4.34 × 10^−2^, Calebin A: adj.*p* = 1.18 × 10^−3^) were enriched in both datasets, aligning with the established lysosomal functions of cathepsins B, Z and A. Additionally, focal adhesion (GO:0005925; obesity: adj.*p* = 4.34 × 10^−2^, Calebin A: adj.*p* = 2.63 × 10^−2^) also showed overlap, suggesting cytoskeletal remodelling (Figure 7b).

The overlap in Molecular Function was limited, with only arachidonate epoxygenase activity (GO:0008392; both adj.*p* ≈ 4.6 × 10^−2^) and arachidonate monooxygenase activity (GO:0008391; both adj.*p* ≈ 4.8 × 10^−2^) achieving significance in both sets, implying potential cross‐talk in lipid mediator metabolism (Figure 7c).

The KEGG pathway analysis revealed significant overlap in axon guidance (hsa04360; obesity: adj.*p* = 1.47 × 10^−3^, Calebin A: adj.*p* = 2.69 × 10^−2^), apoptosis (hsa04210; obesity: adj.*p* = 3.88 × 10^−2^, Calebin A: adj.*p* = 2.85 × 10^−13^) and gap junction (hsa04540; obesity: adj.*p* = 4.19 × 10^−2^, Calebin A: adj.*p* = 2.48 × 10^−6^). The pronounced enrichment of apoptosis within the Calebin A dataset (*p*adj = 2.85 × 10^−13^) aligns with the established pro‐apoptotic properties of curcuminoids (Figure 7d).

The Reactome analysis demonstrated extensive overlap, revealing numerous immune and signalling pathways shared between the two studies. Notable pathways included neutrophil degranulation (obesity: adj.*p* = 1.87 × 10^−3^, Calebin A: adj.*p* = 3.06 × 10^−6^), immune system (obesity: adj.*p* = 1.70 × 10^−2^, Calebin A: adj.*p* = 6.10 × 10^−13^) and axon guidance (obesity: adj.*p* = 1.87 × 10^−2^, Calebin A: adj.*p* = 1.27 × 10^−4^), which were among the most significantly overlapping pathways, thereby underscoring the role of inflammatory and neuro‐guidance mechanisms (Figure 7e). These functional overlaps suggest that Calebin A may affect processes related to obesity at the pathway level, irrespective of a substantial direct gene intersection.

### 3.8 Molecular Docking Analysis

In this study, molecular docking was performed to investigate the binding potential of Calebin‐A against hub proteins overexpressed in obesity. The three‐dimensional structure of Calebin‐A was retrieved from the PubChem database, while the 3D conformations of obesity‐associated hub proteins (CTSB, CTSZ, GRN, CTSA and TUBB) were sourced from the AlphaFold and PDB databases (Table 3). Structural models from AlphaFold exhibited high confidence, with average pLDDT scores ranging from 77.26 (GRN) to 92.3 (CTSZ), validating their reliability for docking analyses.

**TABLE 3:** Three‐dimensional protein structures obtained from the AlphaFold and PDB database.

Protein target	Identifier	METHOD	Average pLDDT^a^	POSITIONS
CTSB	8HEI	X‐ray	1.55 Å	77–333
CTSZ	AF‐Q9UBR2‐F1	AlphaFold	92.3	1–303
GRN	AF‐P28799‐F1	AlphaFold	77.26	1–593
CTSA	4CI9	X‐ray	1.58 Å	29–480
TUBB	AF‐P07437‐F1	AlphaFold	91.79	1–444

^a^ In AlphaFold, the average pLDDT score, which indicates the confidence in the predicted structure, is a measure of the reliability of the predicted atomic positions, with higher values representing greater confidence.

**FIGURE 8:**
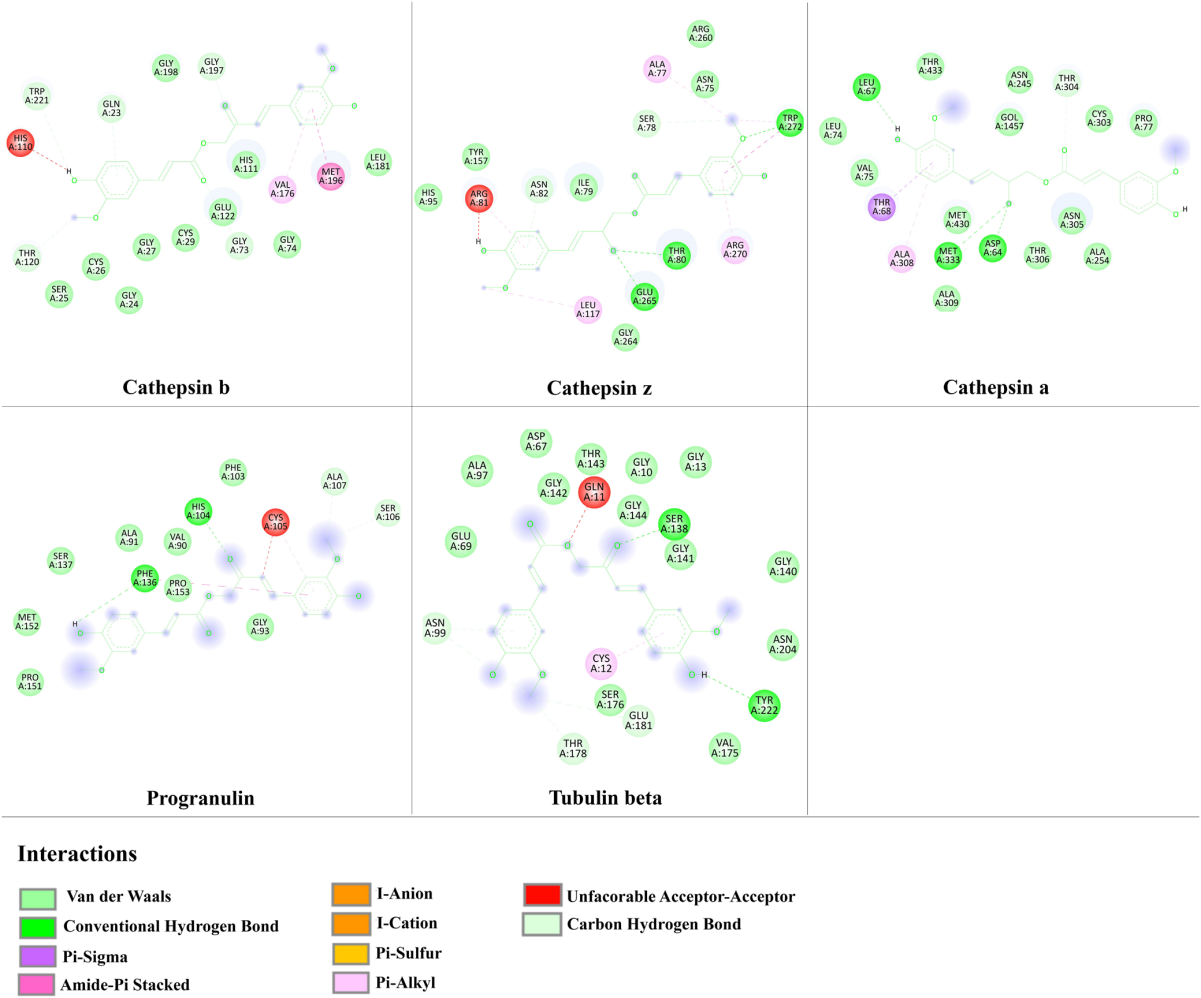
Binding modes of Calebin‐A with central proteins involved in obesity.

The molecular docking results demonstrated robust binding affinities between Calebin‐A and all evaluated hub proteins, as evidenced by favourable Gibbs free energy (ΔGbind) scores (Table 4). Notably, Calebin‐A exhibited the strongest affinity for Cathepsin Z (CTSZ; ΔGbind = −7.1 kcal/mol), followed by Cathepsin A (CTSA; ΔGbind = −6.7 kcal/mol) and Tubulin Beta (TUBB; ΔGbind = −6.5 kcal/mol). These interactions were further stabilized by hydrogen bonds and hydrophobic contacts. For instance, CTSZ engaged in multiple hydrogen bonds with ARG81 (2.18 and 2.16 Å) and GLU265 (2.14 Å) (Table 4, Figure 8). Similarly, TUBB formed hydrogen bonds with SER138 (3.05 Å) and TYR222 (3.18 Å), suggesting a dual‐mode stabilization mechanism.

**TABLE 4:** Hydrogen bond interactions between Calebin‐A and obesity‐associated hub proteins along with Negative ΔGbind values (kcal/mol).

Target	ΔGbind (kcal/mol)	H‐bond interaction	*X* (Å)	*Y* (Å)	*Z* (Å)	Distance (Å)	Donor	Acceptor
Cathepsin B	−6.2	Prot:HIS110:ND1–Lig:O	−14.52	4.42	18.84	3.16	ND1	O
Cathepsin Z	−7.1	Prot:ARG81:HE–Lig:O	4.59	−13.89	−5.88	2.18	HE	O
		Prot:ARG81:HH22–Lig:O	4.61	−14.74	−5.22	2.16	HH22	O
		Prot:GLU265:HN–Lig:O	7.36	−12.46	−13.90	2.14	HN	O
Cathepsin A	−6.7	Cof:NAG1:O6–Lig:O	−3.96	15.23	−41.98	3.03	O6	O
Progranulin	−5.2	Lig:H–Lig:O	5.01	−9.45	30.46	2.11	H	O
Tubulin Beta	−6.5	Prot:SER138:OG–Lig:O	−0.50	−14.55	−2.83	3.05	OG	O
		Prot:TYR222:OH–Lig:O	−1.59	−16.96	−10.61	3.18	OH	O

Abbreviations: ARG, Arginine; ASP, Aspartate; Cof, Cofactor; Distance, Hydrogen bond length (Å); Donor/Acceptor, Atoms involved in the hydrogen bond; GLN, Glutamine; GLU, Glutamate; H‐bond, Hydrogen Bond; HE/HH22, Hydrogens in arginine's side chain; HIS, Histidine; HN, Backbone amide hydrogen; LEU, Leucine; Lig, Calebin‐A; ND1, δ1 nitrogen in histidine; O6, Oxygen position 6 in NAG; OD2, δ2 oxygen in aspartate; OE1, ε1 oxygen in glutamine; OG, γ oxygen in serine; OH, Hydroxyl oxygen in tyrosine; Prot, protein residue; SER, Serine; TYR, Tyrosine; X/Y/Z (Å), Spatial coordinates of the acceptor atom.

A detailed analysis of hydrogen bond interactions (Table 4) revealed that polar residues such as HIS110 (CTSB) and ARG81 (CTSZ) served as primary anchors for Calebin‐A binding. The donor‐acceptor distances for these interactions ranged from 2.11 Å (GRN: Lig:H—Lig:O) to 3.18 Å (TUBB:TYR222:OH), indicative of stable and conformationally favourable bonds. Intriguingly, Calebin‐A also engaged in unconventional interactions, such as a hydrogen bond with the co‐crystallized N‐acetylglucosamine (NAG) moiety in the lysosomal protein (distance: 3.03 Å), underscoring its versatility in targeting diverse binding pockets.

### 3.9 Machine Learning‐Based Enhancement of Docking Predictions

The machine learning‐derived pK values (SVM, RF, consensus) were consistent with the classical. The pK values derived from machine learning (SVM, RF, consensus) aligned well with the traditional ΔG rankings, with CTSZ exhibiting the highest scores (consensus pK = 9.56, SVM pK = 11.91, RF pK = 7.22).

### 3.10 Molecular Dynamics and Binding Energy Evaluation

The protein–ligand complexes with the most favourable docking scores were selected for molecular dynamics simulations, since lower docking energies generally reflect stronger predicted affinity and a higher probability of stable interaction during the simulation period. Based on this ranking, the cathepsin_z complex was chosen for further investigation.

**FIGURE 9:**
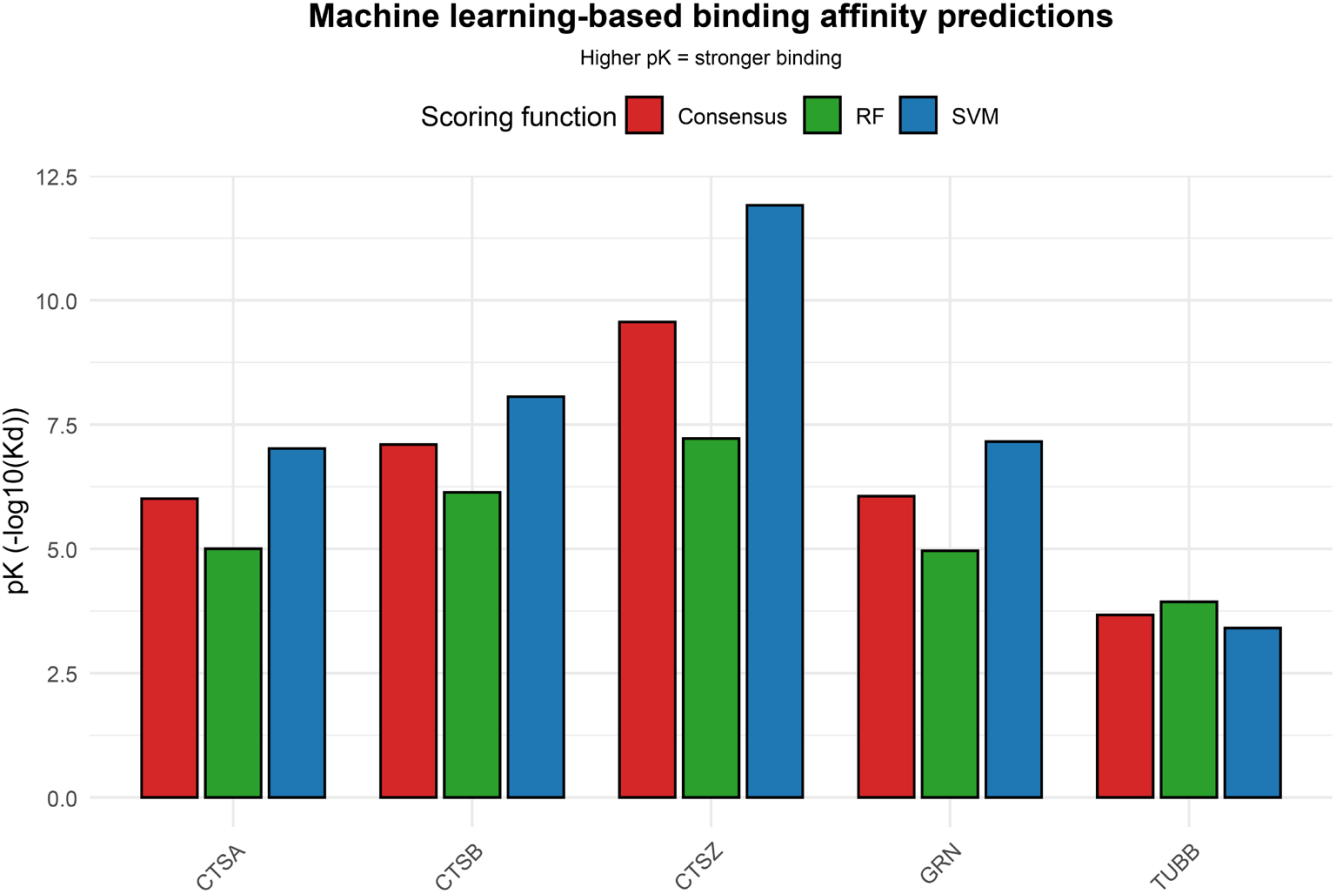
Machine learning‐based on docking predictions. Bar plot showing pK values (higher = stronger binding) derived from three independent scoring functions: Consensus (average of classical functions), SVM (support vector machine) and RF (random forest).

**FIGURE 10:**
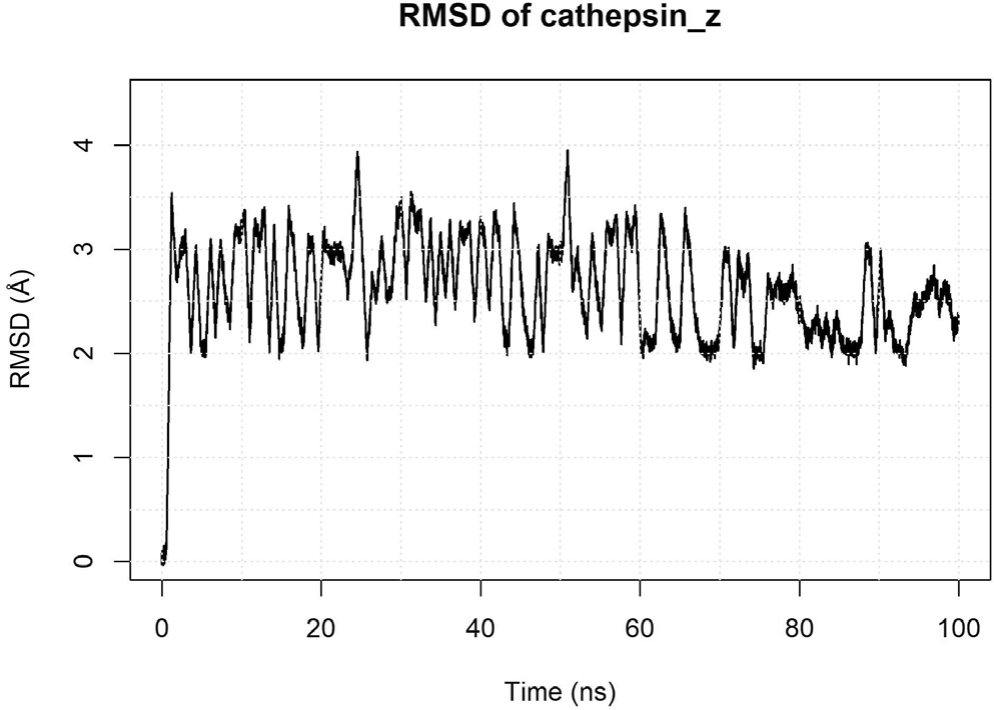
Structural stability and compactness analysis of the cathepsin_z complex during 100 ns of molecular dynamics simulation. The backbone RMSD profile illustrates the equilibration behaviour and overall stability of the complex, confirming its conformational consistency throughout the simulation period.

Analysis of the backbone RMSD profiles showed that the selected complex reached a stable equilibrium within the first few nanoseconds of simulation. For the cathepsin_z complex, RMSD values remained in the range of 2–3.5 Å throughout the first 100 ns, indicating overall structural stability (Figures 9 and 10).

Binding energy decomposition further supported the favourable interaction of the evaluated complexes. Among them, the cathepsin_z complex exhibited the most favourable binding free energy, calculated as −110 ± 7 kJ/mol. This interaction was primarily driven by strong van der Waals (−145 ± 6 kJ/mol) and electrostatic (−310 ± 2 kJ/mol) contributions, whereas polar solvation energy (+100 ± 9 kJ/mol) opposed binding. The nonpolar SASA term provided a smaller stabilizing effect. In addition, the presence of 14–18 hydrogen bonds and compact radius of gyration values of 2.0–2.2 nm supported the formation of a stable protein–ligand complex (Table 5).

**TABLE 5:** Molecular dynamics and binding energy results for the selected complex.

Parameter	Cathepsin_z complex
Docking selection	Lowest docking energy
RMSD behaviour	Stable after initial equilibration
RMSD range	2–3.5 Å
Main flexible region	C‐terminal region
Binding free energy	−110 ± 7 kJ/mol
Van der Waals energy	−145 ± 6 kJ/mol
Electrostatic energy	−310 ± 2 kJ/mol
Polar solvation energy	+100 ± 9 kJ/mol
Nonpolar SASA contribution	Mild stabilizing effect
Hydrogen bonds	14–18
Radius of gyration	2.0–2.2 nm

## 4 Discussion

Our study provides computational evidence for the potential of Calebin‐A as a therapeutic agent in obesity, highlighting its interactions with key genes and pathways involved in the disease.

Our study's findings align with emerging evidence highlighting Calebin‐A's multifaceted role in combating obesity and its associated complications, as demonstrated by its modulation of thermogenesis, inflammation, gut microbiota and lipid metabolism.

The independent validation utilizing GSE59034 verified that CTSB, CTSZ, CTSA and GRN are consistently upregulated in obesity across two distinct adipose depots (omental vs. subcutaneous) and two technological platforms (RNA seq vs. microarray). This consistency robustly supports their biological significance and positions them as viable targets for Calebin A. Crucially, while individual TUBB did not achieve significance, several tubulin beta paralogs (TUBB4B, TUBB2A, TUBB2B, TUBB6) were significantly upregulated, indicating a consistent association of the tubulin beta gene family with obesity.

Research in HFD‐fed mice shows that Calebin‐A supplementation (0.1%–0.5%) for 12 weeks significantly reduces body weight, blood glucose and adipose tissue mass while promoting thermogenesis, as evidenced by improved rectal temperature maintenance during cold exposure [52]. This thermogenic effect is accompanied by a reshaping of the gut microbiota, with enrichment of beneficial bacteria such as *Akkermansia* and *Butyricicoccus*, suggesting a novel mechanism for obesity prevention [52]. Additionally, Calebin‐A ameliorates obesity‐associated hyperglycemia by modulating macrophage polarization, reducing pro‐inflammatory cytokines, and enhancing adiponectin and glucose transporter 4 (GLUT4) expression in adipose tissue, while upregulating hepatic AMP‐activated protein kinase (AMPK) and insulin signalling pathways to improve glycogen storage [53]. In the context of non‐alcoholic fatty liver disease, a frequent obesity comorbidity, Calebin‐A targets critical hub genes, including Tumour Protein P53 (TP53) and Signal Transducer and Activator of Transcription 3 (STAT3), and pathways such as mitogen‐activated protein kinase (MAPK), with molecular docking confirming its high binding affinity to these proteins [54]. Furthermore, Calebin‐A inhibits adipogenesis by suppressing peroxisome proliferator‐activated receptor gamma (PPARγ) and fatty acid synthase, while activating AMPK signalling, leading to reduced hepatic steatosis and serum lipid levels in HFD‐fed mice [25]. These collective findings underscore Calebin‐A's potential as a multi‐target therapeutic agent for obesity, supporting our bioinformatics study.

Our functional enrichment and signalling analyses of differentially expressed genes in obesity patients revealed a multilayered network of biological processes, including immune activation, neurodevelopmental remodelling and mitochondrial dysfunction, all of which contribute to obesity pathogenesis.

One of the most significantly enriched pathways involved axon development and guidance, highlighting the crosstalk between neurodevelopment and metabolic regulation. Sanders et al. demonstrated that maternal obesity impairs vagal and hypothalamic axon development through dysregulation of *BDNF* and *Netrin‐1*, affecting long‐term feeding behaviour [55]. Similarly, Nakanishi et al. emphasized the role of semaphorins and other axon guidance cues in inflammation‐driven metabolic disorders, suggesting that these genes participate in immune‐metabolic convergence [56].

Enrichment in response to chemical stress and immune‐related pathways supports the chronic inflammatory phenotype of obese adipose tissue. Stafeev et al. reported that nutrient excess triggers JNK1/2‐mediated signalling, promoting insulin resistance [57]. Moreover, Wang et al. showed how obesity dysregulates adrenergic and taste receptor signalling, influencing ghrelin secretion and energy homeostasis [58].

Obesity‐related DEGs also enriched GTPase activity regulation, aligning with findings by Gettys et al., who observed altered G‐protein signalling in adipose tissue of obese mice [59]. Veeragandham et al. further showed that RalA, a small GTPase, promotes mitochondrial fragmentation, oxidative stress and lipid accumulation in obesity, suggesting a mechanistic link between cytoskeletal signalling and adipocyte dysfunction [60].

Pathways related to vacuolar lumen and secretory granules were enriched, consistent with morphological studies such as those by Selim, who observed intracellular vacuolization in obese liver tissues [61]. Belgareh‐Touzé et al. also highlighted endosomal dysfunction as a central theme in metabolic derangement [62].

Our analysis identified strong enrichment in ECM organization and focal adhesion pathways, which are critical for adipose expansion and fibrosis. Anguita‐Ruiz et al. reported ECM remodelling genes as biomarkers in childhood obesity [63]. Focal adhesion signalling was further supported by Huang et al. who showed that FTO overexpression promotes myogenesis via *FAK*, *PDGFB* and *RAC2*, tying epigenetic regulation to cellular remodelling [64].

KEGG enrichment revealed antigen processing and presentation as a top pathway. Xiao et al. demonstrated that hypertrophic adipocytes can act as antigen‐presenting cells via MHCII expression and JNK–STAT1 signalling, thereby driving CD4+ T‐cell‐mediated inflammation [65]. This was supported by Majdoubi et al., who showed that *Stat1*‐mediated MHCII expression in adipocytes is required for obesity‐induced inflammation [66], and Chng et al., who identified adaptive immunity as a central contributor to insulin resistance [67]. Consistent with these findings, Deng et al. [68] showed that adipocyte MHCII is functionally active and, together with macrophage MHCII, instigates adipose inflammation, whereas Morris et al. [69] reported that CD40 signalling on adipose tissue macrophages regulates MHCII expression and CD4+ T‐cell expansion in obesity.

Beyond adaptive immunity, innate immune cells play a pivotal role. Neutrophils are now recognized as among the first immune cells infiltrating adipose tissue in obesity, actively contributing to inflammation and metabolic complications [70]. Indeed, increased low‐density neutrophils with an inflammatory gene signature are observed in severe obesity and decrease after bariatric surgery [71, 72]. This aligns closely with our Reactome overlap analysis, which identified neutrophil degranulation as a top overlapping pathway between obesity DEGs and Calebin‐A targets.

Arachidonic acid (AA) metabolism and the cytochrome P450 (CYP) epoxygenase pathway also emerged as significant shared molecular functions. We found overlapping enrichment of arachidonate epoxygenase activity and arachidonate monooxygenase activity. Consistently, Pickens et al. [73] reported that obesity is positively associated with AA‐derived 5‐ and 11‐HETE, while Olona et al. [74] showed that epoxygenase (Cyp2j4) deletion exacerbates diet‐induced adipocyte dysfunction, PPARγ downregulation and hepatic steatosis. Wang et al. [75] demonstrated that CYP‐derived fatty acid epoxides are the most dramatically reduced lipid mediators in adipose tissue of obese mice, and Zhao et al. [76] found decreased epoxygenase and increased soluble epoxide hydrolase expression in mesenteric arteries of obese rats. Moreover, Zhang et al. [77] linked distinct gut microbiota and arachidonic acid metabolism to obesity‐prone versus obesity‐resistant phenotypes, and Xu et al. [78] showed that arachidonic acid inhibits angiotensin‐converting enzyme in human adipocytes via an NF‐κB‐dependent pathway.

In this work, network‐based integration of transcriptomic data from obese omental adipose tissue highlighted several key hubs upregulated in obesity. Molecular docking further predicted high‐affinity binding of the phytochemical calebin‐A to each protease, suggesting potential multi‐target modulation of these genes.

CTSB has been extensively implicated in obesity‐associated lysosomal dysfunction and pro‐inflammatory signalling. Araujo et al. [79] systematically reviewed how nutrient excess in WAT drives CTSB release, excessive autophagic flux, macrophage infiltration and metabolic derangements such as insulin resistance in obese models. Complementing this, gene‐expression analysis in human subcutaneous adipose tissue revealed that decreased CTSB mRNA correlates with markers of insulin resistance independent of adiposity, indicating a direct link between CTSB dysregulation and impaired glucose homeostasis [80]. At the protein level, Mizunoe et al. [81] demonstrated that CTSB overexpression in 3T3‐L1 adipocytes induces perilipin 1 degradation, disrupting lipid‐droplet integrity and impairing lipolysis, key features of obese WAT dysfunction. Beyond adipocytes, CTSB blockade with Ca‐074Me abolished palmitate‐induced Nlrp3 inflammasome activation, tight‐junction disruption, and increased endothelial permeability in microvascular endothelial cells, linking CTSB to vascular complications of obesity such as early endothelial injury [82]. Finally, in macrophages and adipocytes, CTSB (and CTSL) regulate tumour necrosis factor alpha (TNF‐α) production, NPC2 secretion and cholesterol‐trafficking gene expression, underscoring its role in inflammation and lipid handling [83]. Our docking model predicts that calebin‐A forms a hydrogen bond with CTSB His110 (ΔG_bind = −6.2 kcal/mol), potentially acting as a competitive inhibitor to rebalance autophagy and curb inflammatory signalling.

While CTSZ has received less direct attention in obesity, it clusters functionally with other papain‐like proteases. In obese humans, Naour et al. found that cathepsin S (CTSS) mRNA and release increase twofold in WAT and 30% in circulation, whereas CTSL remains unchanged and CTSK is undetectable, highlighting selective regulation within the family [84]. In abdominal SCAT, reduced CTSL and by extension perhaps CTSZ expression marks lysosomal dysfunction and correlates with insulin resistance [80]. In vitro, CTSL and CTSB secretion from adipocytes and macrophages responds to TNF‐α and Lipopolysaccharide (LPS), modulating inflammatory outputs and NPC2 secretion [83]. Although CTSZ's specific role in obesity remains to be delineated, its structural similarity to CTSB/L suggests it may contribute to extracellular‐matrix remodelling, immune‐cell recruitment and lipid‐droplet turnover in hypertrophic WAT. Notably, calebin‐A showed even stronger docking to CTSZ (hydrogen bonds with Arg81 and Glu265; ΔG_bind = −7.1 kcal/mol), indicating potential to attenuate CTSZ‐mediated tissue remodelling.

Cathepsin A (CTSA), or protective protein (PPCA), emerged as an obesity‐associated hub, upregulated in omental fat. Hiraiwa first characterized CTSA's multifunctional lysosomal roles including carboxypeptidase, deamidase and esterase activities and its protective complex formation with β‐galactosidase and neuraminidase in lysosomes [85]. Cuervo et al. [86] later showed that PPCA cleaves the CMA receptor LAMP2a, controlling CMA flux: loss of PPCA elevates LAMP2a levels and CMA rates, while rescue of PPCA restores normal CMA. More recently, Sun et al. demonstrated that anti‐obesity cyclic peptides inhibit CTSA–Neu1 interaction, leading to perilipin 1 degradation and reduced fat accumulation, revealing CTSA as a modulable node in adipocyte lipid handling [87]. In a different context, leptin downregulates CTSA in canine mammary adenocarcinoma cells, upregulating LAMP2a and CMA activity, underscoring CTSA's influence on cell survival pathways in obesity‐related conditions [88]. In our docking, calebin‐A engages CTSA's N‐acetylglucosamine–binding site (ΔG_bind = −6.7 kcal/mol), suggesting it may fine‐tune CTSA's dual protease–chaperone functions to restore healthy CMA and proteostasis in obese WAT.

Taken together, CTSB, CTSZ and CTSA drive complementary aspects of lysosomal dysfunction in obesity: excessive autophagy and inflammation (CTSB), extracellular‐matrix remodelling and cell trafficking (CTSZ) and dysregulated CMA/proteostasis (CTSA). By binding predicted active‐site and regulatory residues across all three proteases with sub‐micromolar affinity, calebin‐A holds promise as a multi‐target modulator. This polypharmacology may rebalance lipid‐droplet stability, curb inflammatory cytokine release, preserve matrix integrity and normalize autophagic flux.

Multiple studies have established PGRN as an adipose‐derived cytokine whose circulating levels are elevated in obesity and T2D. Korolczuk [89] highlighted that hyperprogranulinemia contributes to insulin resistance by impairing insulin signalling and decreasing glucose uptake both in vitro and in vivo. Moreover, PGRN deficiency in mice protected against diet‐induced insulin resistance, implicating this protein as a causative factor in the metabolic dysregulation of obesity [89].

In other research, Qu et al. [90] provided direct clinical evidence from Chinese cohorts, demonstrating that plasma PGRN levels were significantly elevated in obese and diabetic patients, with strong correlations to BMI, waist circumference, triglycerides, HbA1c, IL‐6 and HOMA‐IR [90]. In line with these findings, Youn et al. [91] demonstrated that PGRN serum concentrations correlate with macrophage infiltration in omental adipose tissue, C‐reactive protein (CRP), and total cholesterol. They also showed that physical exercise significantly reduced circulating PGRN in type 2 diabetic patients, confirming that PGRN is dynamically regulated by metabolic state and serves as a marker of visceral inflammation [91].

Moreover, Alissa et al. [55] reported that children in the upper quartile of PGRN concentrations had higher insulin resistance, dyslipidemia and systemic inflammation, supporting its role as a metabolic biomarker from early life stages [55]. However, Niklowitz et al. [92] found no strong association between PGRN and insulin resistance or metabolic syndrome in a 2‐year intervention study, suggesting age‐dependent or context‐specific regulatory mechanisms.

In reproductive health, Gorkem et al. [93] found that serum PGRN levels were elevated in obese infertile women, correlating with BMI and testosterone.

In addition, Brock et al. [94] conducted a large‐scale human study comparing subcutaneous and visceral adipose tissue (AT) PGRN expression in bariatric surgery patients. They found that serum PGRN levels were not directly predicted by AT gene expression, despite detectable expression in both depots. Interestingly, serum levels rose markedly post‐surgery, suggesting extra‐adipose sources or altered clearance mechanisms may contribute to circulating PGRN regulation in obesity [94].

Our docking results revealed a stable binding interaction between calebin‐A and PGRN, suggesting that this compound may interfere with its pro‐inflammatory or metabolic signalling. This echoes prior work with curcumin, a structurally related phytochemical with well‐documented anti‐inflammatory effects mediated by various targets in different disease/pathological states [95, 96, 97].

For instance, curcumin treatment alleviated inflammation and keratinocyte hyperproliferation in PGRN‐deficient mice with imiquimod‐induced psoriatic lesions. Notably, curcumin attenuated IL‐17A expression and epidermal thickening, indicating a direct anti‐inflammatory role that could be extrapolated to other PGRN‐related pathologies, including obesity [98].

In our systems‐level analysis of obesity‐associated gene networks, tubulin beta (TUBB) was identified as a significant hub node in the omental adipose tissue interactome (degree = 8; betweenness = 279.1). TUBB was upregulated in obesity samples, and docking simulations revealed that calebin‐A binds TUBB with a moderate affinity (ΔG_bind = −6.4 kcal/mol), forming a hydrogen bond with Asn101, suggesting the potential for bioactive modulation of TUBB's function.

TUBB encodes a key component of microtubules, which are critical for a wide range of cellular processes including intracellular trafficking, glucose transporter translocation (e.g., GLUT4), vesicle transport and cell division [55]. The interaction between microtubules and the linker of the nucleoskeleton complex is essential for the adipogenic differentiation of human adipose‐derived stem cells [99].

Genetic studies have begun to unravel TUBB's role in metabolic pathophysiology. Liu et al. [100] demonstrated that specific TUBB single nucleotide polymorphisms (SNPs), including rs3132584 and rs2222896, were significantly associated with dyslipidemia risk in the Chinese Maonan ethnic group. Certain TUBB haplotypes increased susceptibility to low HDL and high LDL cholesterol levels, suggesting that tubulin β variants can influence lipid homeostasis and may contribute to the development of obesity‐related dyslipidemia [100].

Complementary evidence from Liu et al. in 2022 linked TUBB SNPs (e.g., rs3130685, rs2278075) and their interactions with WWOX and EHBP1 to the risk of coronary artery disease and ischemic stroke. Notably, these interactions were also influenced by lifestyle and environmental factors such as diabetes, hypertension and alcohol consumption, implying that TUBB functions at a critical interface between genetic susceptibility and metabolic behaviour [101].

Recent findings have expanded the mechanistic links between tubulin family members and metabolic dysfunction. Luo et al. [102] identified TUBB as a core gene in NAFLD‐associated hepatocellular carcinoma, showing its diagnostic and prognostic value, and demonstrated that targeting TUBB inhibits cancer cell proliferation, migration and invasion—processes that also parallel adipocyte expansion and adipose tissue remodelling in obesity [102]. Moreover, a de novo mutation in TUBB (p.G308S) was shown to impair ciliogenesis by altering microtubule dynamics and structure [103]. Since primary cilia are critical for adipocyte differentiation, insulin signalling and energy homeostasis, impaired ciliogenesis could directly contribute to obesity pathogenesis. Indeed, Tong et al. [104] identified rare loss‐of‐function variants in the TUB gene (a related tubulin family member) in young‐onset obesity patients [104]. The homologous TUB p.R363G mutation led to hyperphagia, leptin resistance, and impaired AgRP neuron response in mice, demonstrating that tubulin‐related proteins can influence central appetite regulation and energy balance [104].

Liotti et al. [105] reported that periprostatic adipose tissue promotes docetaxel resistance in prostate cancer via upregulation of the β‐tubulin isoform TUBB2B, mediated by paracrine IGF‐1 signalling. This finding underscores the importance of adipose tissue‐derived factors in modulating tubulin expression and function, which may also affect metabolic tissues in obesity [105]. Collectively, these studies indicate that tubulin family members, including TUBB, are not only structural proteins but also active players in lipid metabolism, insulin and leptin signalling, ciliary function and adipose–organ crosstalk.

Expanding beyond metabolic disorders, Stoupa et al. [106] identified TUBB1 mutations as causative factors in thyroid dysgenesis. Mutated β1‐tubulin proteins were unable to integrate into microtubules, disrupting thyroid organogenesis and hormone production. These findings not only expand the role of tubulin β isoforms in endocrine development but also point to broader implications for hormonal regulation and systemic metabolic signalling in the context of obesity [106].

Given the centrality of thyroid hormones in basal metabolic rate regulation, TUBB dysfunction may contribute to adiposity and lipid disorders via disrupted thyroid physiology. While our current focus is on class I β‐tubulin (TUBB), these findings underscore the relevance of tubulin family proteins in both structural cellular dynamics and endocrine–metabolic axes.

Several limitations must be taken into account when analysing the validation results. Firstly, the discovery dataset (GSE286454) and the validation dataset (GSE59034) differ in terms of adipose tissue depot, specifically omental versus subcutaneous. Secondly, the methodologies employed were not the same: RNA sequencing was used for discovery while microarray technology was utilized for validation, potentially leading to variability across the datasets. Thirdly, the sample sizes were significantly different (*n* = 5 per group in discovery compared to *n* = 16 per group in validation), and the limited size of the discovery cohort heightens the likelihood of false positives or unstable effect size estimates. As a result, the incomplete validation of TUBB at the individual gene level may be indicative of these biological and technical variances rather than a genuine lack of association. Nonetheless, the consistent upregulation of the tubulin beta family (TUBB4B, TUBB2A, TUBB2B, TUBB6) in the validation dataset reinforces the broader biological significance of these pathways. Future research involving larger, adequately powered and depot‐matched cohorts is essential to validate the roles of these gene families in obesity and to assess their modulation by Calebin A.

## 5 Conclusion

In this research, we employed a comprehensive approach that integrates transcriptomics, network analysis, and structural biology to explore the efficacy of Calebin‐A in the context of obesity. The examination of RNA‐seq data from adipose tissue revealed significant obesity‐related differentially expressed genes, which facilitated the construction of a protein–protein interaction (PPI) network. This network allowed us to identify and prioritize essential hubs, such as lysosomal proteases (CTSZ, CTSB, CTSA), GRN and TUBB. Furthermore, pathway enrichment analysis and comparisons with predicted targets of Calebin‐A indicated a convergence on pathways associated with lysosomal function, immune response, apoptosis and cytoskeletal dynamics that are pertinent to adipose tissue in obesity. Molecular docking studies, combined with machine learning‐based scoring and 100 ns molecular dynamics simulations utilizing MM‐PBSA calculations, demonstrated a notably stable interaction between Calebin‐A and CTSZ. Collectively, our in silico results imply that Calebin‐A has the potential to influence critical molecular pathways associated with obesity, underscoring the importance of CTSZ and related lysosomal‐immune mechanisms as viable targets for subsequent experimental validation and the development of Calebin‐A as a potential adjunctive approach in the management of obesity.

## Author Contributions

**Ali Mahmoudi:** conceptualization, investigation, writing – original draft. **Ali Saeedi‐Boroujeni:** investigation, writing – original draft. **Sercan Karav:** investigation, writing – review and editing. **Prashant Kesharwani:** investigation, writing – review and editing, supervision. **Amirhossein Sahebkar:** conceptualization, investigation, writing – review and editing, supervision.

## Funding

This study was supported by Abadan University of Medical Sciences under Grant No. 2312 and approved by the Institutional Ethics Committee (Ethics Code: IR.ABADANUMS.REC.1404.111).

## Conflicts of Interest

The authors declare no conflicts of interest.

## Data Availability

Authors elect not to share the data.

## References

[1] S. Wharton , D. C. Lau , M. Vallis , et al., “Obesity in Adults: A Clinical Practice Guideline,” CMAJ 192, no. 31 (2020): E875–E891. 32753461 10.1503/cmaj.191707 PMC7828878

[2] L. Busetto , S. Bettini , J. Makaronidis , C. A. Roberts , J. C. Halford , and R. L. Batterham , “Mechanisms of Weight Regain,” European Journal of Internal Medicine 93 (2021): 3–7. 33461826 10.1016/j.ejim.2021.01.002

[3] J. B. Echouffo‐Tcheugui and E. Selvin , “Prediabetes and What It Means: The Epidemiological Evidence,” Annual Review of Public Health 42, no. 1 (2021): 59–77. 10.1146/annurev-publhealth-090419-102644 PMC8026645 33355476

[4] L. Perreault , Q. Pan , K. J. Mather , K. E. Watson , R. F. Hamman , and S. E. Kahn , “Effect of Regression From Prediabetes to Normal Glucose Regulation on Long‐Term Reduction in Diabetes Risk: Results From the Diabetes Prevention Program Outcomes Study,” Lancet 379, no. 9833 (2012): 2243–2251. 22683134 10.1016/S0140-6736(12)60525-X PMC3555407

[5] D. Kinlen , D. Cody , and D. O'Shea , “Complications of Obesity,” QJM: An International Journal of Medicine 111, no. 7 (2018): 437–443. 29025162 10.1093/qjmed/hcx152

[6] J. Lu , P. Liu , M. Cai , et al., “Recent Progress in the Pharmacotherapy for Obesity,” European Journal of Pharmacology 1002 (2025): 177850. 40553780 10.1016/j.ejphar.2025.177850

[7] W. Ren , Y. Xia , S. Chen , et al., “Glutamine Metabolism in Macrophages: A Novel Target for Obesity/Type 2 Diabetes,” Advances in Nutrition 10, no. 2 (2019): 321–330. 30753258 10.1093/advances/nmy084 PMC6416106

[8] I. A.‐O. X. Caruso , A. A.‐O. Cignarelli , G. A.‐O. Sorice , S. A.‐O. Perrini , and F. A.‐O. Giorgino , “Incretin‐Based Therapies for the Treatment of Obesity‐Related Diseases” (2948‐2828 (Electronic)). 10.1038/s44324-024-00030-5 PMC12118674 40604322

[9] E. Melson , U. Ashraf , D. Papamargaritis , and M. J. Davies , “What Is the Pipeline for Future Medications for Obesity?,” International Journal of Obesity 49, no. 3 (2025): 433–451. 38302593 10.1038/s41366-024-01473-y PMC11971045

[10] W. T. Garvey , “New Horizons. A New Paradigm for Treating to Target With Second‐Generation Obesity Medications,” Journal of Clinical Endocrinology & Metabolism 107, no. 4 (2022): e1339–e1347. 34865050 10.1210/clinem/dgab848 PMC8947217

[11] A. A. Tahrani and J. Morton , “Benefits of Weight Loss of 10% or More in Patients With Overweight or Obesity: A Review,” Obesity 30, no. 4 (2022): 802–840. 35333446 10.1002/oby.23371

[12] D. E. Arterburn , E. Johnson , K. J. Coleman , et al., “Weight Outcomes of Sleeve Gastrectomy and Gastric Bypass Compared to Nonsurgical Treatment,” Annals of Surgery 274, no. 6 (2021): e1269–e1276. 32187033 10.1097/SLA.0000000000003826

[13] V. L. Gloy , M. Briel , D. L. Bhatt , et al., “Bariatric Surgery Versus Non‐Surgical Treatment for Obesity: A Systematic Review and Meta‐Analysis of Randomised Controlled Trials,” BMJ 347 (2013): 347. 10.1136/bmj.f5934 PMC3806364 24149519

[14] L. M. Carlsson , M. Peltonen , S. Ahlin , et al., “Bariatric Surgery and Prevention of Type 2 Diabetes in Swedish Obese Subjects,” New England Journal of Medicine 367, no. 8 (2012): 695–704. 22913680 10.1056/NEJMoa1112082

[15] J. Wardle , S. Carnell , C. M. Haworth , and R. Plomin , “Evidence for a Strong Genetic Influence on Childhood Adiposity Despite the Force of the Obesogenic Environment,” American Journal of Clinical Nutrition 87, no. 2 (2008): 398–404. 18258631 10.1093/ajcn/87.2.398

[16] R. Mahmoud , V. Kimonis , and M. G. Butler , “Genetics of Obesity in Humans: A Clinical Review,” International Journal of Molecular Sciences 23, no. 19 (2022): 11005. 36232301 10.3390/ijms231911005 PMC9569701

[17] D. S. H. L. Kim and J. Y. Kim , “Total Synthesis of Calebin‐A, Preparation of Its Analogues, and Their Neuronal Cell Protectivity Against β‐Amyloid Insult,” Bioorganic & Medicinal Chemistry Letters 11, no. 18 (2001): 2541–2543. 11549465 10.1016/s0960-894x(01)00489-9

[18] A. Majeed , M. Majeed , N. Thajuddin , et al., “Bioconversion of Curcumin Into Calebin‐A by the Endophytic Fungus Ovatospora Brasiliensis EPE‐10 MTCC 25236 Associated With *Curcuma caesia* ,” AMB Express 9, no. 1 (2019): 79. 31144200 10.1186/s13568-019-0802-9 PMC6541684

[19] A. L. Oliveira , S. E. Martinez , K. Nagabushnam , et al., “Calebin A: Analytical Development for Pharmacokinetics Study, Elucidation of Pharmacological Activities and Content Analysis of Natural Health Products,” Journal of Pharmacy & Pharmaceutical Sciences 18, no. 4 (2015): 494–514. 26626247 10.18433/j32310

[20] H. M. M. Arafa , R. A. Hemeida , A. I. M. El‐Bahrawy , and F. M. A. Hamada , “Prophylactic Role of Curcumin in Dextran Sulfate Sodium (DSS)‐Induced Ulcerative Colitis Murine Model,” Food and Chemical Toxicology 47, no. 6 (2009): 1311–1317. 19285535 10.1016/j.fct.2009.03.003

[21] A. L. Cheng , C. H. Hsu , J. K. Lin , et al., “Phase I Clinical Trial of Curcumin, a Chemopreventive Agent, in Patients With High‐Risk or Pre‐Malignant Lesions,” Anticancer Research 21, no. 4b (2001): 2895–2900. 11712783

[22] A. Nair , A. Amalraj , J. Jacob , A. B. Kunnumakkara , and S. Gopi , “Non‐Curcuminoids From Turmeric and Their Potential in Cancer Therapy and Anticancer Drug Delivery Formulations,” Biomolecules 9, no. 1 (2019): 13. 30609771 10.3390/biom9010013 PMC6358877

[23] A. Mahmoudi , P. Kesharwani , M. Majeed , Y. Teng , and A. Sahebkar , “Recent Advances in Nanogold as a Promising Nanocarrier for Curcumin Delivery,” Colloids and Surfaces B: Biointerfaces 215 (2022): 112481. 35453063 10.1016/j.colsurfb.2022.112481

[24] M. Majeed , K. Nagabhushanam , S. Natarajan , S. Bani , A. Pandey , and S. K. Karri , “Investigation of Repeated Dose (90 Day) Oral Toxicity, Reproductive/Developmental Toxicity and Mutagenic Potential of ‘Calebin A’,” Toxicology Reports 2 (2015): 580–589. 28962393 10.1016/j.toxrep.2015.03.009 PMC5598428

[25] C. S. Lai , S. N. Liao , M. L. Tsai , et al., “Calebin‐A Inhibits Adipogenesis and Hepatic Steatosis in High‐Fat Diet‐Induced Obesity via Activation of AMPK Signaling,” Molecular Nutrition & Food Research 59, no. 10 (2015): 1883–1895. 26108684 10.1002/mnfr.201400809

[26] A. Oulas , G. Minadakis , M. Zachariou , K. Sokratous , M. M. Bourdakou , and G. M. Spyrou , “Systems Bioinformatics: Increasing Precision of Computational Diagnostics and Therapeutics Through Network‐Based Approaches,” Briefings in Bioinformatics 20, no. 3 (2019): 806–824. 29186305 10.1093/bib/bbx151 PMC6585387

[27] X. Meng , J. Fau ‐ Xiang , J. Xiang , et al., “DPCMNE: Detecting Protein Complexes From Protein‐Protein Interaction Networks via Multi‐Level Network Embedding” (1557‐9964 (Electronic)). 10.1109/TCBB.2021.3050102 33417563

[28] J. A.‐O. Xiang , J. Zhang , R. Zheng , X. Li , and M. A.‐O. Li , “NIDM: Network Impulsive Dynamics on Multiplex Biological Network for Disease‐Gene Prediction,” LID – bbab080 [pii] LID (1477‐4054 (Electronic)), 10.1093/bib/bbab080. 33866352

[29] R. Zhou , Z. Lu , H. Luo , J. Xiang , M. Zeng , and M. Li , “NEDD: A Network Embedding Based Method for Predicting Drug‐Disease Associations” (1471‐2105 (Electronic)). 10.1186/s12859-020-03682-4 PMC7495830 32938396

[30] Y. Qian , Q. Wang , L. Yin , and A. A.‐O. Lu , “CF‐DTI: Coarse‐To‐Fine Feature Extraction for Enhanced Drug‐Target Interaction Prediction” (2047‐2501 (Print)). 10.1007/s13755-025-00370-6 PMC12408429 40919585

[31] A. Mahmoudi , S. Heydari , Y. V. Markina , G. E. Barreto , and A. Sahebkar , “Role of Statins in Regulating Molecular Pathways Following Traumatic Brain Injury: A System Pharmacology Study,” Biomedicine & Pharmacotherapy 153 (2022): 113304. 35724514 10.1016/j.biopha.2022.113304

[32] A. Mahmoudi , S. L. Atkin , N. G. Nikiforov , and A. Sahebkar , “Therapeutic Role of Curcumin in Diabetes: An Analysis Based on Bioinformatic Findings,” Nutrients 14, no. 15 (2022): 3244. 35956419 10.3390/nu14153244 PMC9370108

[33] Y. A.‐O. Wu , L. A.‐O. Yang , X. A.‐O. Wu , et al., “Identification of the Hub Genes in Polycystic Ovary Syndrome Based on Disease‐Associated Molecule Network” (1530‐6860 (Electronic)). 10.1096/fj.202202103R 37342921

[34] Y. A.‐O. Tai , H. Tian , X. Yang , et al., “Identification of Hub Genes and Candidate Herbal Treatment in Obesity Through Integrated Bioinformatic Analysis and Reverse Network Pharmacology” (2045‐2322 (Electronic)). 10.1038/s41598-022-22112-4 PMC9556576 36224334

[35] B. Gayakvad and M. Misra , “Network Pharmacology and In‐Silico Pharmacological Insights Into the Anti‐Obesity Potential of *Madhuca longifolia* Flowers,” Pharmacological Research – Natural Products 9 (2025): 100444.

[36] E. Hu , Z. Li , T. Li , et al., “A Novel Microbial and Hepatic Biotransformation‐Integrated Network Pharmacology Strategy Explores the Therapeutic Mechanisms of Bioactive Herbal Products in Neurological Diseases: The Effects of Astragaloside IV on Intracerebral Hemorrhage as an Example,” Chinese Medicine 18, no. 1 (2023): 40. 37069580 10.1186/s13020-023-00745-5 PMC10108474

[37] M. Cheng , T. Li , E. Hu , et al., “A Novel Strategy of Integrating Network Pharmacology and Transcriptome Reveals Antiapoptotic Mechanisms of Buyang Huanwu Decoction in Treating Intracerebral Hemorrhage” (1872‐7573 (Electronic)). 10.1016/j.jep.2023.117123 37673200

[38] A. Mahmoudi , A. E. Butler , M. Majeed , M. Banach , and A. Sahebkar , “Investigation of the Effect of Curcumin on Protein Targets in NAFLD Using Bioinformatic Analysis,” Nutrients 14, no. 7 (2022): 1331. 35405942 10.3390/nu14071331 PMC9002953

[39] C. Mao , T. D. Howard , D. Sullivan , et al., “Bioinformatic Analysis of Coronary Disease Associated SNPs and Genes to Identify Proteins Potentially Involved in the Pathogenesis of Atherosclerosis,” Journal of Proteomics and Genomics Research 2, no. 1 (2017): 1–12. 29367937 10.14302/issn.2326-0793.jpgr-17-1447 PMC5777528

[40] A. Mahmoudi , A. E. Butler , M. Banach , T. Jamialahmadi , and A. Sahebkar , “Identification of Potent Small‐Molecule PCSK9 Inhibitors Based on Quantitative Structure‐Activity Relationship, Pharmacophore Modeling, and Molecular Docking Procedure,” Current Problems in Cardiology 48 (2023): 101660. 36841313 10.1016/j.cpcardiol.2023.101660

[41] L. Priscilla , K. Viol Dhea , A. Arif Nur Muhammad , et al., “In Silico Phytochemical Compounds Screening of *Allium sativum* Targeting the Mpro of SARS‐CoV‐2,” Pharmacognosy Journal 14, no. 3 (2022): 604–609.

[42] A. Nur Sofiatul , K. Viol Dhea , W. Muhammad Hermawan , et al., “In Silico Screening of Bioactive Compounds From *Syzygium cumini* L. and *Moringa oleifera* L. Against SARS‐CoV‐2 via Tetra Inhibitors,” Pharmacognosy Journal 14, no. 4 (2022): 267–272.

[43] A. R. Melge , K. Manzoor , S. V. Nair , and C. G. Mohan , “In Silico Modeling of FDA‐Approved Drugs for Discovery of Anti‐Cancer Agents: A Drug‐Repurposing Approach,” in In Silico Drug Design (Elsevier, 2019), 577–608.

[44] A. Mahmoudi , S. L. Atkin , T. Jamialahmadi , M. Banach , and A. Sahebkar , “Effect of Curcumin on Attenuation of Liver Cirrhosis via Genes/Proteins and Pathways: A System Pharmacology Study,” Nutrients 14, no. 20 (2022): 4344. 36297027 10.3390/nu14204344 PMC9609422

[45] M. V. Kuleshov , M. R. Jones , A. D. Rouillard , et al., “Enrichr: A Comprehensive Gene Set Enrichment Analysis Web Server 2016 Update,” Nucleic Acids Research 44, no. W1 (2016): W90–W97. 27141961 10.1093/nar/gkw377 PMC4987924

[46] S. Kim , J. Chen , T. Cheng , et al., “PubChem in 2021: New Data Content and Improved Web Interfaces,” Nucleic Acids Research 49, no. D1 (2021): D1388–D1395. 33151290 10.1093/nar/gkaa971 PMC7778930

[47] P. K. Sharma and I. S. Yadav , “Biological Databases and Their Application,” in Bioinformatics (Elsevier, 2022), 17–31.

[48] S. Dallakyan and A. J. Olson , “Small‐Molecule Library Screening by Docking With PyRx,” in Chemical Biology (Springer, 2015), 243–250. 10.1007/978-1-4939-2269-7_19 25618350

[49] R. Wang , Y. Lu , and S. Wang , “Comparative Evaluation of 11 Scoring Functions for Molecular Docking,” Journal of Medicinal Chemistry 46, no. 12 (2003): 2287–2303. 12773034 10.1021/jm0203783

[50] G. B. Li , L. L. Yang , W. J. Wang , L. L. Li , and S. Y. Yang , “ID‐Score: A New Empirical Scoring Function Based on a Comprehensive Set of Descriptors Related to Protein‐Ligand Interactions,” Journal of Chemical Information and Modeling 53, no. 3 (2013): 592–600. 23394072 10.1021/ci300493w

[51] P. J. Ballester and J. B. Mitchell , “A Machine Learning Approach to Predicting Protein‐Ligand Binding Affinity With Applications to Molecular Docking,” Bioinformatics 26, no. 9 (2010): 1169–1175. 20236947 10.1093/bioinformatics/btq112 PMC3524828

[52] P.‐S. Lee , Y.‐Y. Lu , K. Nagabhushanam , C.‐T. Ho , H.‐C. Mei , and M.‐H. Pan , “Calebin‐A Prevents HFD‐Induced Obesity in Mice by Promoting Thermogenesis and Modulating Gut Microbiota,” Journal of Traditional and Complementary Medicine 13, no. 2 (2023): 119–127. 36970457 10.1016/j.jtcme.2022.01.001 PMC10037069

[53] C. Anwar , J.‐R. Lin , M.‐L. Tsai , C.‐T. Ho , and C.‐S. Lai , “Calebin A Attenuated Inflammation in RAW264.7 Macrophages and Adipose Tissue to Improve Hepatic Glucose Metabolism and Hyperglycemia in High‐Fat Diet‐Fed Obese Mice,” European Journal of Pharmacology 978 (2024): 176789. 38945287 10.1016/j.ejphar.2024.176789

[54] A. Mahmoudi , M. M. Hajihasani , M. Majeed , T. Jamialahmadi , and A. Sahebkar , “Effect of Calebin‐A on Critical Genes Related to NAFLD: A Protein‐Protein Interaction Network and Molecular Docking Study,” Current Genomics 25, no. 2 (2024): 120–139. 38751599 10.2174/0113892029280454240214072212 PMC11092913

[55] E. M. Alissa , R. H. Sutaih , H. Z. Kamfar , A. E. Alagha , and Z. M. Marzouki , “Serum Progranulin Levels in Relation to Insulin Resistance in Childhood Obesity,” Journal of Pediatric Endocrinology and Metabolism 30, no. 12 (2017): 1251–1256. 29176029 10.1515/jpem-2017-0321

[56] Y. Nakanishi , S. Kang , and A. Kumanogoh , “Axon Guidance Molecules in Immunometabolic Diseases,” Inflammation and Regeneration 42, no. 1 (2022): 5. 35045890 10.1186/s41232-021-00189-0 PMC8767680

[57] I. Stafeev , S. Michurina , N. Podkuychenko , M. Menshikov , Y. V. Parfyonova , and A. Vorotnikov , “Chemical Inducers of Obesity‐Associated Metabolic Stress Activate Inflammation and Reduce Insulin Sensitivity in 3T3‐L1 Adipocytes,” Biochemistry (Moscow) 84 (2019): 553–561. 31234769 10.1134/S0006297919050092

[58] Q. Wang , K. I. Liszt , E. Deloose , et al., “Obesity Alters Adrenergic and Chemosensory Signaling Pathways That Regulate Ghrelin Secretion in the Human Gut,” FASEB Journal 33, no. 4 (2019): 4907–4920. 30629462 10.1096/fj.201801661RR

[59] T. Gettys , V. Ramkumar , R. Uhing , L. Seger , and I. Taylor , “Alterations in mRNA Levels, Expression, and Function of GTP‐Binding Regulatory Proteins in Adipocytes From Obese Mice (C57BL/6J‐ob/ob),” Journal of Biological Chemistry 266, no. 24 (1991): 15949–15955. 1908462

[60] P. Veeragandham , Y. Cao , Y. Xu , et al., “Obesity‐Dependent Increase in RalA Activity Disrupts Mitochondrial Dynamics in White Adipocytes,” Research Square (2023): rs.3.rs‐2923510.

[61] S. A. Selim , “The Effect of High‐Fat Diet‐Induced Obesity on the Parotid Gland of Adult Male Albino Rats: Histological and Immunohistochemical Study,” Egyptian Journal of Histology 36, no. 4 (2013): 772–780.

[62] N. Belgareh‐Touzé , S. Avaro , Y. Rouillé , B. Hoflack , and R. Haguenauer‐Tsapis , “Yeast Vps55p, a Functional Homolog of Human Obesity Receptor Gene‐Related Protein, Is Involved in Late Endosome to Vacuole Trafficking,” Molecular Biology of the Cell 13, no. 5 (2002): 1694–1708. 12006663 10.1091/mbc.01-12-0597 PMC111137

[63] A. Anguita‐Ruiz , M. Bustos‐Aibar , J. Plaza‐Díaz , et al., “Omics Approaches in Adipose Tissue and Skeletal Muscle Addressing the Role of Extracellular Matrix in Obesity and Metabolic Dysfunction,” International Journal of Molecular Sciences 22, no. 5 (2021): 2756. 33803198 10.3390/ijms22052756 PMC7963192

[64] H. Huang , L. Liu , C. Li , et al., “Fat Mass‐ and Obesity‐Associated (FTO) Gene Promoted Myoblast Differentiation Through the Focal Adhesion Pathway in Chicken,” 3 Biotech 10 (2020): 1–10. 32903970 10.1007/s13205-020-02386-z PMC7447686

[65] L. Xiao , X. Yang , Y. Lin , et al., “Large Adipocytes Function as Antigen‐Presenting Cells to Activate CD4+ T Cells via Upregulating MHCII in Obesity,” International Journal of Obesity 40, no. 1 (2016): 112–120. 26248660 10.1038/ijo.2015.145 PMC4722243

[66] A. Majdoubi , O. A. Kishta , and J. Thibodeau , “Role of Antigen Presentation in the Production of Pro‐Inflammatory Cytokines in Obese Adipose Tissue,” Cytokine 82 (2016): 112–121. 26854212 10.1016/j.cyto.2016.01.023

[67] M. H. Y. Chng , M. N. Alonso , S. E. Barnes , K. D. Nguyen , and E. G. Engleman , “Adaptive Immunity and Antigen‐Specific Activation in Obesity‐Associated Insulin Resistance,” Mediators of Inflammation 2015, no. 1 (2015): 593075. 26146464 10.1155/2015/593075 PMC4471324

[68] T. Deng , C. J. Lyon , L. J. Minze , et al., “Class II Major Histocompatibility Complex Plays an Essential Role in Obesity‐Induced Adipose Inflammation,” Cell Metabolism 17, no. 3 (2013): 411–422. 23473035 10.1016/j.cmet.2013.02.009 PMC3619392

[69] D. L. Morris , K. E. Oatmen , T. A. Mergian , et al., “CD40 Promotes MHC Class II Expression on Adipose Tissue Macrophages and Regulates Adipose Tissue CD4+ T Cells With Obesity,” Journal of Leukocyte Biology 99, no. 6 (2016): 1107–1119. 26658005 10.1189/jlb.3A0115-009R PMC4952010

[70] E. Uribe‐Querol and C. Rosales , “Neutrophils Actively Contribute to Obesity‐Associated Inflammation and Pathological Complications,” Cells 11, no. 12 (2022): 1883. 35741012 10.3390/cells11121883 PMC9221045

[71] M. D. Sanchez‐Pino , W. S. Richardson , J. Zabaleta , et al., “Increased Inflammatory Low‐Density Neutrophils in Severe Obesity and Effect of Bariatric Surgery: Results From Case‐Control and Prospective Cohort Studies,” eBioMedicine 77 (2022): 103910. 35248994 10.1016/j.ebiom.2022.103910 PMC8897585

[72] X. Xu , S. Su , X. Wang , et al., “Obesity Is Associated With More Activated Neutrophils in African American Male Youth,” International Journal of Obesity 39, no. 1 (2015): 26–32. 25388404 10.1038/ijo.2014.194 PMC4286492

[73] C. A. Pickens , L. M. Sordillo , C. Zhang , and J. I. Fenton , “Obesity Is Positively Associated With Arachidonic Acid‐Derived 5‐ and 11‐Hydroxyeicosatetraenoic Acid (HETE),” Metabolism 70 (2017): 177–191. 28403941 10.1016/j.metabol.2017.01.034

[74] A. Olona , X. Terra , J.‐H. Ko , et al., “Epoxygenase Inactivation Exacerbates Diet and Aging‐Associated Metabolic Dysfunction Resulting From Impaired Adipogenesis,” Molecular Metabolism 11 (2018): 18–32. 29656108 10.1016/j.molmet.2018.03.003 PMC6001407

[75] W. Wang , J. Yang , W. Qi , et al., “Lipidomic Profiling of High‐Fat Diet‐Induced Obesity in Mice: Importance of Cytochrome P450‐Derived Fatty Acid Epoxides,” Obesity 25, no. 1 (2017): 132–140. 27891824 10.1002/oby.21692 PMC5182168

[76] X. Zhao , A. Dey , O. P. Romanko , et al., “Decreased Epoxygenase and Increased Epoxide Hydrolase Expression in the Mesenteric Artery of Obese Zucker Rats,” American Journal of Physiology. Regulatory, Integrative and Comparative Physiology 288, no. 1 (2005): R188–R196. 15345471 10.1152/ajpregu.00018.2004

[77] J. Zhang , K. Z. Sanidad , and G. Zhang , “Cytochrome P450 Monooxygenase/Soluble Epoxide Hydrolase‐Mediated Eicosanoid Pathway in Colorectal Cancer and Obesity‐Associated Colorectal Cancer,” Oncoscience 6, no. 9–10 (2019): 371–375. 31763369 10.18632/oncoscience.488 PMC6855364

[78] L. Xu , R. Schüler , C. Xu , et al., “Arachidonic Acid Inhibits the Production of Angiotensin‐Converting Enzyme in Human Primary Adipocytes via a NF‐κB‐Dependent Pathway,” Annals of Translational Medicine 8, no. 24 (2020): 1652. 33490164 10.21037/atm-20-7514 PMC7812212

[79] T. F. Araujo , A. V. Cordeiro , D. A. Vasconcelos , K. F. Vitzel , and V. R. Silva , “The Role of Cathepsin B in Autophagy During Obesity: A Systematic Review,” Life Sciences 209 (2018): 274–281. 30107168 10.1016/j.lfs.2018.08.024

[80] Q. Xu , E. C. M. Mariman , G. H. Goossens , E. E. Blaak , and J. W. E. Jocken , “Cathepsin Gene Expression in Abdominal Subcutaneous Adipose Tissue of Obese/Overweight Humans,” Adipocytes 9, no. 1 (2020): 246–252. 10.1080/21623945.2020.1775035 PMC7469552 32486882

[81] Y. Mizunoe , M. Kobayashi , S. Hoshino , et al., “Cathepsin B Overexpression Induces Degradation of Perilipin 1 to Cause Lipid Metabolism Dysfunction in Adipocytes,” Scientific Reports 10, no. 1 (2020): 634. 31959889 10.1038/s41598-020-57428-6 PMC6971249

[82] L. Wang , Y. Chen , X. Li , Y. Zhang , E. Gulbins , and Y. Zhang , “Enhancement of Endothelial Permeability by Free Fatty Acid Through Lysosomal Cathepsin B‐Mediated Nlrp3 Inflammasome Activation,” Oncotarget 7, no. 45 (2016): 73229–73241. 27689324 10.18632/oncotarget.12302 PMC5341975

[83] J. Hannaford , H. Guo , and X. Chen , “Involvement of Cathepsins B and L in Inflammation and Cholesterol Trafficking Protein NPC2 Secretion in Macrophages,” Obesity 21, no. 8 (2013): 1586–1595. 23666609 10.1002/oby.20136 PMC6445554

[84] N. Naour , C. Rouault , S. Fellahi , et al., “Cathepsins in Human Obesity: Changes in Energy Balance Predominantly Affect Cathepsin s in Adipose Tissue and in Circulation,” Journal of Clinical Endocrinology & Metabolism 95, no. 4 (2010): 1861–1868. 20164293 10.1210/jc.2009-1894

[85] M. Hiraiwa , “Cathepsin A/Protective Protein: An Unusual Lysosomal Multifunctional Protein,” Cellular and Molecular Life Sciences 56 (1999): 894–907. 11212324 10.1007/s000180050482 PMC11146757

[86] A. M. Cuervo , L. Mann , E. J. Bonten , A. d'Azzo , and J. F. Dice , “Cathepsin A Regulates Chaperone‐Mediated Autophagy Through Cleavage of the Lysosomal Receptor,” EMBO Journal 22 (2003): 47–59. 12505983 10.1093/emboj/cdg002 PMC140041

[87] Y. Sun , A. Dakiiwa , M. Zhang , T. Shibata , and M. Kita , “Inhibition of Lysosomal Cathepsin A and Neuraminidase 1 Interaction by Anti‐Obesity Cyclic Peptide,” Chemistry – A European Journal 30, no. 56 (2024): e202402049. 39115037 10.1002/chem.202402049

[88] J.‐W. Kim , F. Y. Mahiddine , and G. A. Kim , “Leptin Modulates the Metastasis of Canine Inflammatory Mammary Adenocarcinoma Cells Through Downregulation of Lysosomal Protective Protein Cathepsin A (CTSA),” International Journal of Molecular Sciences 21, no. 23 (2020): 8963. 33255835 10.3390/ijms21238963 PMC7728357

[89] A. Korolczuk , “Progranulin, a New Adipokine at the Crossroads of Metabolic Syndrome, Diabetes, Dyslipidemia and Hypertension,” Current Pharmaceutical Design 23, no. 10 (2017): 1533–1539. 28120721 10.2174/1381612823666170124114524

[90] H. Qu , H. Deng , and Z. Hu , “Plasma Progranulin Concentrations Are Increased in Patients With Type 2 Diabetes and Obesity and Correlated With Insulin Resistance,” Mediators of Inflammation 2013, no. 1 (2013): 360190. 23476101 10.1155/2013/360190 PMC3588183

[91] B.‐S. Youn , S.‐I. Bang , N. Kloting , et al., “Serum Progranulin Concentrations May Be Associated With Macrophage Infiltration Into Omental Adipose Tissue,” Diabetes 58, no. 3 (2009): 627–636. 19056610 10.2337/db08-1147 PMC2646061

[92] P. Niklowitz , J. Rothermel , N. Lass , A. Barth , and T. Reinehr , “Is There a Link Between Progranulin, Obesity, and Parameters of the Metabolic Syndrome in Children? Findings From a Longitudinal Intervention Study,” Pediatric Diabetes 20, no. 8 (2019): 1047–1055. 31469472 10.1111/pedi.12915

[93] U. Gorkem , Z. O. Inal , H. A. Inal , and M. O. Bostanci , “Serum Progranulin Levels Are Elevated in Infertile Women With Obesity,” Endokrynologia Polska 69, no. 6 (2018): 661–666. 30229554 10.5603/EP.a2018.0044

[94] J. Brock , A. Schmid , T. Karrasch , et al., “Progranulin Serum Levels and Gene Expression in Subcutaneous vs Visceral Adipose Tissue of Severely Obese Patients Undergoing Bariatric Surgery,” Clinical Endocrinology 91, no. 3 (2019): 400–410. 31102282 10.1111/cen.14040

[95] M. Saberi‐Karimian , M. Keshvari , M. Ghayour‐Mobarhan , et al., “Effects of Curcuminoids on Inflammatory Status in Patients With Non‐Alcoholic Fatty Liver Disease: A Randomized Controlled Trial,” Complementary Therapies in Medicine 49 (2020): 102322. 32147075 10.1016/j.ctim.2020.102322

[96] M. Sadeghi , S. Dehnavi , A. Asadirad , et al., “Curcumin and Chemokines: Mechanism of Action and Therapeutic Potential in Inflammatory Diseases,” Inflammopharmacology 31, no. 3 (2023): 1069–1093. 36997729 10.1007/s10787-023-01136-w PMC10062691

[97] H. S. Shamsnia , M. Roustaei , D. Ahmadvand , et al., “Impact of Curcumin on p38 MAPK: Therapeutic Implications” (1568‐5608 (Electronic)). 10.1007/s10787-023-01265-2 37498375

[98] T. Zhou , S. Zhang , Y. Zhou , et al., “Curcumin Alleviates Imiquimod‐Induced Psoriasis in Progranulin‐Knockout Mice,” European Journal of Pharmacology 909 (2021): 174431. 34428436 10.1016/j.ejphar.2021.174431

[99] A. Sferra , S. Petrini , E. Bellacchio , et al., “TUBB Variants Underlying Different Phenotypes Result in Altered Vesicle Trafficking and Microtubule Dynamics,” International Journal of Molecular Sciences 21, no. 4 (2020): 1385. 32085672 10.3390/ijms21041385 PMC7073044

[100] C.‐X. Liu , R.‐X. Yin , Z.‐H. Shi , et al., “Associations Between TUBB‐WWOX SNPs, Their Haplotypes, Gene‐Gene, and Gene‐Environment Interactions and Dyslipidemia,” Aging (Albany NY) 13, no. 4 (2021): 5906–5927. 33612478 10.18632/aging.202514 PMC7950260

[101] C.‐X. Liu , R.‐X. Yin , X.‐L. Cao , et al., “EHBP1, TUBB, and WWOX SNPs, Gene‐Gene and Gene‐Environment Interactions on Coronary Artery Disease and Ischemic Stroke,” Frontiers in Genetics 13 (2022): 843661. 35559044 10.3389/fgene.2022.843661 PMC9086287

[102] Z. Luo , Y. Zhang , C. Guo , et al., “Integrated Bioinformatics and In Vitro Validation Reveal TUBB as a Core Gene and Therapeutic Target in NAFLD‐Related Hepatocellular Carcinoma,” International Journal of Biological Macromolecules 334, no. Pt 2 (2025): 149026. 41271048 10.1016/j.ijbiomac.2025.149026

[103] A. Mollica , S. Omer , G. Forguson , et al., “Mutations in the β‐Tubulin TUBB Impair Ciliogenesis and Are Associated With Ciliopathy‐Like Phenotypes,” Nature Communications 16, no. 1 (2025): 10637. 10.1038/s41467-025-65634-x PMC12660950 41309602

[104] M. Tong , Y. Chen , B. Song , et al., “The TUB Variant Impairs Leptin Sensitivity and AgRP Neuronal Response, Leading to Obesity,” Science Translational Medicine 18, no. 836 (2026): eadw0458. 41671341 10.1126/scitranslmed.adw0458

[105] A. Liotti , E. La Civita , M. Cennamo , et al., “Periprostatic Adipose Tissue Promotes Prostate Cancer Resistance to Docetaxel by Paracrine IGF‐1 Upregulation of TUBB2B Beta‐Tubulin Isoform,” Prostate 81, no. 7 (2021): 407–417. 33734457 10.1002/pros.24117 PMC8251776

[106] A. Stoupa , F. Adam , D. Kariyawasam , et al., “TUBB 1 Mutations Cause Thyroid Dysgenesis Associated With Abnormal Platelet Physiology,” EMBO Molecular Medicine 10, no. 12 (2018): e9569. 30446499 10.15252/emmm.201809569 PMC6284387

